# Beyond Plasmonics: Au Nanoparticles as Electron Sinks in TiO_2_ for Interface Passivation Enhancement in Planar Perovskite Solar Cells

**DOI:** 10.1021/acsami.5c24570

**Published:** 2026-01-28

**Authors:** Diogo F. Carvalho, Pedro Conceição, Andrés D. Pardo Perdomo, Ricardo Silva, Manuel Martins, Jennifer P. Teixeira, Pedro M. P. Salomé, Paulo Fernandes, Maria Rosário Correia

**Affiliations:** † 246702 INL—International Iberian Nanotechnology Laboratory, Braga 4715-330, Portugal; ‡ i3N—Institute for Nanostructures, Nanomodelling and Nanofabrication, 56062 University of Aveiro, Aveiro 3810-193, Portugal; § Department of Physics, University of Aveiro, Aveiro 3810-193, Portugal; ∥ CICECO, University of Aveiro, Aveiro 3810-193, Portugal; ⊥ Instituto de Energía Solar, ETSI Telecomunicación, Universidad Politécnica de Madrid, Madrid 28040, Spain; # PCI - Creative Science Park, Ílhavo 3830-352, Portugal; ¶ Department of Physics, ISEP—Porto School of Engineering, CIETI, Porto 4249-015, Portugal

**Keywords:** perovskite solar cells, plasmonics, gold nanoparticles, electron transport layer, TiO_2_, interface passivation, electron-sink effect, device stability

## Abstract

Improving the interface passivation and charge selectivity of electron transport layers (ETLs) is essential to enhance both performance and operational stability in perovskite solar cells (PSCs). In this work, we introduce a sputtered double compact TiO_2_ ETL incorporating monodisperse Au nanoparticles (NPs) as an electronically active interlayer. The sputtering process ensures conformal encapsulation of the NPs and precise control of TiO_2_ sublayer thickness, providing a highly controlled platform to disentangle structural, optical, and electronic effects of embedded metal NPs. This architecture enables, for the first time, a systematic investigation of an electron-sink-induced field-effect modulation of the AuNPs@TiO_2_/perovskite interface. By precisely controlling NP size (15 and 55 nm), loading (0.15–1.20 wt %), and TiO_2_ encapsulation thickness (5–20 nm), we identified an optimized architecture that delivers a 1.3% absolute gain in light-to-power conversion efficiency, primarily through increased short-circuit current density. Structural and optical analyses confirm that NPs are uniformly embedded without modifying the TiO_2_ crystallinity and electronic structure. The results indicate that embedded Au NPs may passivate interface traps and enhance ETL selectivity, while their electron-sink behavior transiently captures charge and modulates the interface potential. At high NP concentrations (>0.30 wt %), overlapping depletion regions lead to transport constrictions and increased transport resistance. Furthermore, the Au NPs appear to suppress the UV-driven photocatalytic activity of TiO_2_, improving device operational stability. These findings reveal a new electron-sink mechanism in metal-oxide/metal-NP systems and establish sputtered AuNPs@TiO_2_ ETLs as a scalable route toward more selective and stable perovskite photovoltaics.

## Graphical abstract

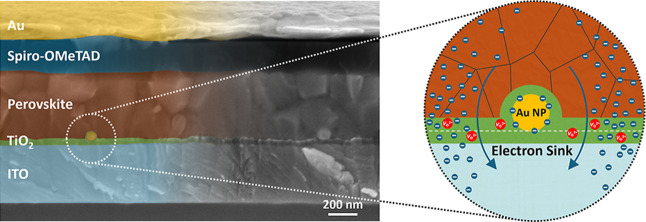

## 1 Introduction

Metal halide perovskite solar cells (PSCs) have emerged as one of the most promising photovoltaic technologies of the past decade, combining the solution-processability^1^ and low manufacturing costs^2^ of thin-film semiconductors with light-to-power conversion efficiency (PCE) values rivaling those of crystalline silicon.^3^ Their exceptional optoelectronic properties,^4^ including high absorption coefficient, long carrier diffusion lengths, tunable band gap, and defect tolerance, have enabled certified PCE values exceeding 26%.^5–7^ Moreover, the compatibility of perovskite materials with scalable low-temperature deposition techniques,^8,9^ and flexible substrates^10^ makes them highly attractive for large-area manufacturing and tandem integration. Nevertheless, the practical deployment of PSCs remains challenged by interface losses^11^ and stability issues,^12^ particularly at the transport layers, where charge extraction and recombination processes compete and degradation reactions are more likely to occur.^13^

In particular, the electron transport layer (ETL) plays a crucial role in defining device performance. It must efficiently extract photogenerated electrons from the perovskite absorber while blocking holes, to minimize interface recombination.^14^ Titanium dioxide (TiO_2_) is one of the most established ETLs due to its suitable band alignment, transparency, and chemical stability.^15,16^ However, its relatively low conductivity,^17^ the presence of deep trap states,^18^ and its UV-induced photocatalytic activity^19,20^ can limit both the efficiency and the long-term durability of PSCs.

Several strategies have been explored to overcome these issues, including chemical doping,^21–23^ interface passivation,^24–27^ and incorporation of plasmonic nanoparticles (NPs).^28–35^ In particular, noble metal NPs, such as Au and Ag, have been incorporated into PSCs to enhance performance through a combination of optical and electronic mechanisms.^36^ Optically, localized surface plasmons (LSPs) in metallic NPs can enhance the local electromagnetic field and increase light scattering, thereby improving light harvesting within the perovskite absorber. However, when the NPs are embedded within compact ETLs rather than inside the perovskite bulk, the optical field enhancement is strongly screened, making purely plasmonic contributions less effective.^37,38^ Instead, several electronic mechanisms have been proposed to explain the observed performance variations. These include (i) hot-electron injection from metal NPs into the ETL conduction band,^29^ (ii) improved transport of charge carriers,^28,31,35^ (iii) trap passivation at the ETL/perovskite interface,^39^ and (iv) modulation of electronic band bending and Fermi-level alignment,^40^ which influence interface recombination and transport dynamics. Despite these multiple possibilities, most prior studies^31,35,41^ dispersed NPs in solution-processed ETLs or at ill-defined interfaces, often lacking precise control over NP density, distribution, and encapsulation; factors that critically determine the dominant mechanism and device reproducibility.

Here, we report a sputtered double compact TiO_2_ ETL incorporating monodisperse spherical Au NPs of controlled size and density between the two sublayers, enabling precise control of the TiO_2_/Au architecture. The sputtering process ensures conformal encapsulation of the NPs and precise control of each TiO_2_ sublayer’s thickness, allowing for a systematic and reproducible study of the structural, optical, and electronic effects induced by embedded Au NPs in a highly controlled TiO_2_ platform. Beyond plasmonic contributions, a combination of impedance spectroscopy, photoluminescence, and intensity-dependent *J*–*V* analyses uncovers a previously unreported electronic mechanism: embedded Au NPs act as electron-sink centers that transiently accumulate charge and modulate the TiO_2_/perovskite interfacial potential through a capacitive, depletion-like effect. Such charge capture and storage phenomena are well-known in photocatalytic TiO_2_-metal systems^42–44^ but have not previously been explored in perovskite solar cells or connected to their photovoltaic response and stability. This dynamic charge buffering enhances charge selectivity, beyond interface passivation. The embedded Au NPs further mitigate the UV-driven photocatalytic activity of TiO_2_, thereby suppressing interface degradation pathways and improving the short-term stability of photovoltaic performance. Overall, this study introduces a scalable, sputter-compatible ETL design and provides new insights into how embedded metal NPs can modulate charge dynamics in PSCs through coupled optical, electronic, and electrostatic effects beyond traditional plasmonic mechanisms.

## 2 Experimental Section

### 2.1 Au NP Preparation

All chemicals were purchased from Sigma-Aldrich and used as received without further purification. Au NPs with an average diameter of 15 nm were synthesized via the Turkevish method.^45^ Briefly, 94 mL of a 318 μM HAuCl_4_ aqueous solution was brought to boiling (100 °C) under vigorous stirring, and 667 μL of 170 mM sodium citrate was quickly added. The reaction was maintained at 100 °C for 30 min, after which the total volume was adjusted to 120 mL (corresponding to 250 μM Au). Au NPs with an average diameter of 55 nm were synthesized via a premixing Tris-assisted method previously reported.^46^ A premixing solution was prepared by adding 85 μL of 5.9 mM AgNO_3_ to 1.0 mL of 25.3 mM HAuCl_4_, under stirring. Then, 1.0 mL of 33.9 mM sodium citrate was added to the mixture while stirring. Water was added to adjust the total volume to 3.0 mL, and the solution was kept without stirring for 4 min. Subsequently, 4.0 mL of a 0.10 M Tris­(hydroxymethyl)­aminomethane (Tris) buffer was added under stirring. After 1 min, the premixing solution was quickly injected into 93 mL of boiling water under vigorous stirring. The reaction was maintained at 100 °C for 30 min. For solvent exchange and surface functionalization, 5.0 mL of the as-synthesized Au NP colloidal solutions were mixed with 109 and 397 μL of a 2.6 mM PVP-10 solution for the 15 and 55 nm Au NPs, respectively. The mixtures were stirred for 24 h. The NPs were then precipitated by centrifugation (10,000 rpm for 30 min for the 15 nm NPs, and 5000 rpm for 10 min for the 55 nm NPs) and redispersed in ethanol for further use.

### 2.2 Device Fabrication

All chemicals were purchased from Sigma-Aldrich and used as received without further purification. Commercial ITO-coated glass substrates (2.5 × 2.5 cm^2^) were sequentially cleaned in an ultrasonic bath with soap solution, acetone, isopropanol, and water, each for 15 min. The substrates were then dried under a nitrogen flow and treated with UV–ozone for 15 min. TiO_2_ films were deposited on the ITO substrates by RF magnetron sputtering (Ultra high-vacuum Kenosistec system) at 60 W, using a TiO_2_ target. Colloidal Au NP solution in ethanol (125 μM) was then spin-coated onto the TiO_2_ layer at 2000 rpm for 15 s, followed by a 5 min drying step at 70 °C. To increase the surface coverage of Au NPs, multiple deposition cycles were performed. A second TiO_2_ layer was subsequently deposited again by RF sputtering, after a 15 min UV–ozone treatment, to encapsulate the Au NPs. The samples were then annealed at 500 °C for 30 min in air. After cooling to room temperature, the AuNPs@TiO_2_ substrates were exposed again to UV–ozone for 15 min before being transferred into a nitrogen-filled glovebox. The perovskite precursor solution was prepared by dissolving 0.507 g of PbI_2_, 0.073 g of PbBr_2_, 0.022 g of methylammonium bromide (MABr), 0.172 g of formamidinium iodide (FAI), and 0.019 g of CsI in a solvent mixture of 800 μL dimethylformamide (DMF) and 200 μL dimethyl sulfoxide (DMSO),^47^ achieving a nominal composition of Cs_0.06_MA_0.16_FA_0.78_PbI_2.5_Br_0.5_. A volume of 100 μL of this solution was spin-coated in a two-step program: 1000 rpm for 10 s, followed by 6000 rpm for 30 s. 100 μL of chlorobenzene was dripped onto the spinning substrate 15 s before the end of the second step. The films were then annealed at 100 °C for 40 min. The Spiro-OMeTAD solution was prepared by dissolving 91 mg Spiro-OMeTAD in 1 mL chlorobenzene, followed by the addition of 36 μL of 4-*tert*-butylpyridine, 21 μL of a Li-TSFI solution (104 mg in 200 μL of acetonitrile), and 9 μL of a FK209 solution (80 mg in 200 μL of acetonitrile).^47^ A volume of 80 μL of the solution was spin-coated at 4000 rpm for 15 s. Finally, a 100 nm Au electrode was thermally evaporated on top of the devices under high vacuum.

### 2.3 Characterization

Scanning electron microscopy (SEM) images were obtained using a Nova NanoSEM 650 equipment, operated at a 5 kV acceleration voltage. Transmission electron microscopy (TEM) images were acquired using a JEOL JEM-2100 with an acceleration voltage of 200 kV, with NPs deposited on a carbon-coated Cu grid. Optical absorbance and transmittance were measured using a PerkinElmer Lambda 950 UV–visible spectrophotometer, operated with an integrating sphere. Ellipsometry measurements were performed in a J.A. Wollam M-2000 equipment. X-ray photoelectron spectroscopy (XPS) and ultraviolet photoelectron spectroscopy (UPS) measurements were conducted in an ESCALAB 250Xi system. The XPS spectra were acquired using a Mg Kα X-ray source, and the binding energy scale was calibrated to the C 1s peak at 284.8 eV. The UPS spectra were acquired with the He I source, and the binding-energy calibration was confirmed using an Au sample. Micro-Raman measurements were conducted using a Horiba Jobin-Yvon HR800 spectrometer equipped with a 600 grooves mm^–1^ grating, and a 442 nm He–Cd laser’s excitation line was focused on the top of the samples using a 100× microscope objective. X-ray diffraction (XRD) was performed in a PANalytical X’Pert PRO MRD equipment (Cu Kα X-ray tube, λ = 0.154 nm, 45 kV, and 40 mA). The *J*–*V* curves were measured using a Keithley 2420 SourceMeter and a Newport Oriel Sol3A solar simulator under standard simulated AM 1.5G illumination at room temperature, using a 0.15 cm^2^ mask, at 25 mV/s. External quantum efficiency (EQE) was measured using a Newport QuantX 300 with a mechanical optical chopper at 10 Hz. Impedance and capacitance–voltage measurements were performed using a Keysight E4980A LCR Meter. The impedance frequency was measured from 20 Hz to 2 MHz. The amplitude of the AC signal was 25 mV. Capacitance–voltage measurements were performed for 10 kHz, and the Mott–Schottky analysis included corrections for geometric capacitance and series resistance. Steady-state and time-resolved photoluminescence (PL) were measured on a PicoQuant FluoTime 300 fluorescence lifetime spectrometer, equipped with a picosecond pulsed laser diode with an excitation wavelength of 453.6 nm.

## 3 Results and Discussion

A schematic illustration of the PSC architecture used in this study is shown in Figure 1a. The device adopts a conventional n-i-p planar configuration, comprising the following layers: a transparent indium tin oxide (ITO) contact (thickness ≈400 nm), a double compact TiO_2_ electron transport layer (35 nm) incorporating Au NPs between the two sublayers, a Cs_0.06_MA_0.16_FA_0.78_PbI_2.5_Br_0.5_ triple-cation perovskite absorber (500 nm), a Spiro-OMeTAD hole transport layer (250 nm), and an Au contact (100 nm). The thickness of the compact TiO_2_ layer was optimized by fabricating devices with values ranging from 10 to 40 nm. As shown in Figure S1 (Supporting Information), the PCE increases with TiO_2_ thickness and stabilizes at values ≥35 nm. Therefore, a total thickness of 35 nm for the double compact TiO_2_ layer was selected for all subsequent devices. The absolute efficiencies observed here fall within the typical range of planar devices based on sputtered compact TiO_2_,^48–51^ which was deliberately selected as a highly controlled model ETL with embedded Au NPs, rather than to maximize device performance.

**Figure 1:**
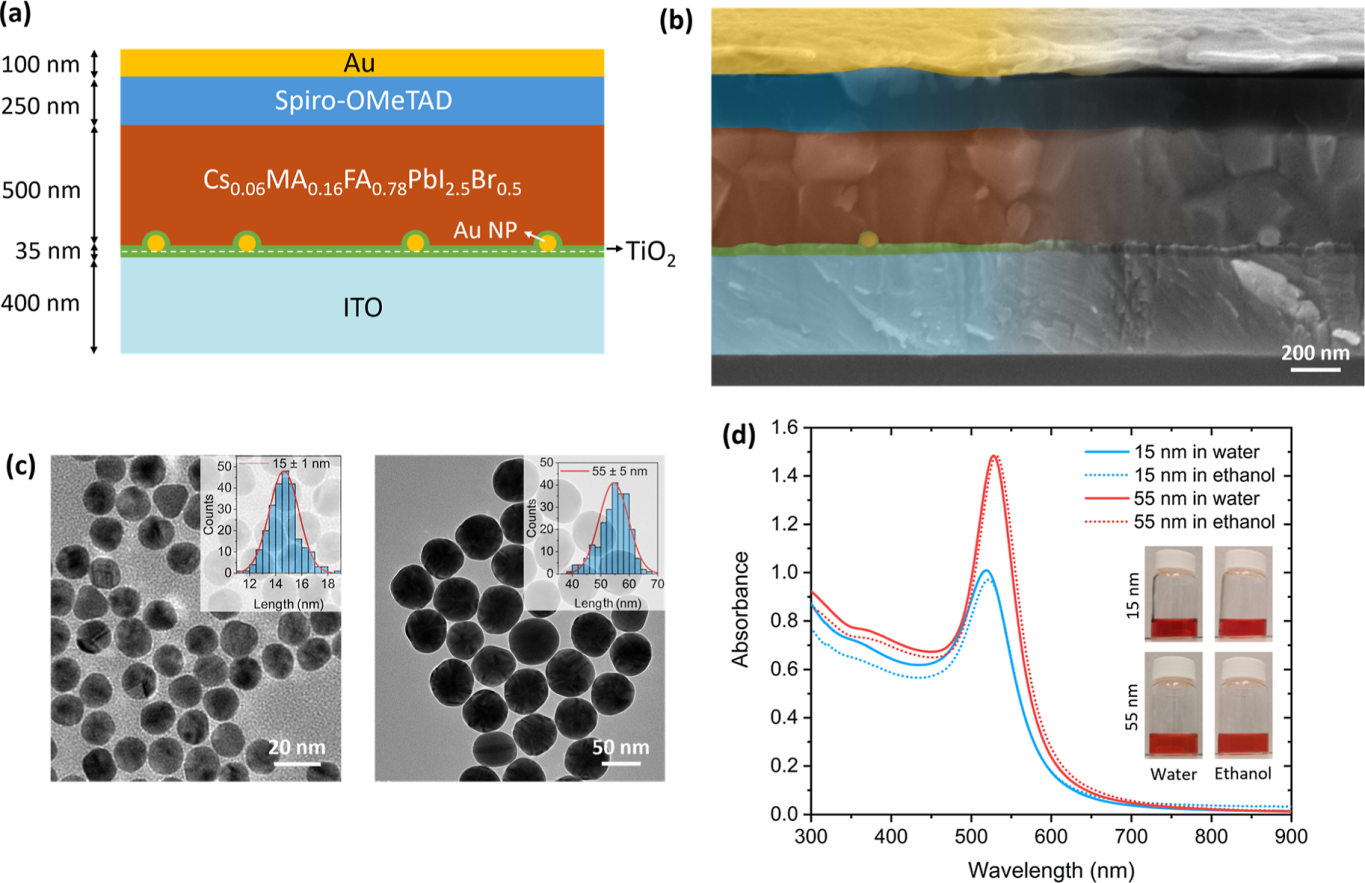
(a) Schematic illustration of the PSC structure. (b) Cross-sectional SEM image of the perovskite with 55 nm diameter Au NPs incorporated between the double TiO_2_ layer. (c) TEM images of the Au NPs with 15 nm (left) and 55 nm (right) diameters, and size distribution histograms (inset). (d) Absorbance spectra of Au NPs in water and ethanol.

Figure 1b shows a cross-sectional SEM image of a device incorporating Au NPs (55 nm diameter) within the double compact TiO_2_ layer. In particular, this device has a 15 nm-thick top TiO_2_ sublayer, while the bottom sublayer is 20 nm-thick. The image reveals that the NPs are encapsulated between the two TiO_2_ sublayers, confirming their successful incorporation into the ETL. Figure 1c displays TEM images of the two Au NP sizes investigated, with diameters of 15 ± 1 nm and 55 ± 5 nm. The choice of these two diameters was intended to probe possible size-dependent optical effects: 15 nm NPs introduce parasitic absorption with negligible scattering, whereas 55 nm NPs provide stronger scattering efficiency, potentially increasing the optical path length in the absorber.^52^ NPs exhibit good sphericity and narrow size distribution, as evidenced by the uniform shapes and sizes. The corresponding optical absorption spectra are presented in Figure 1d. The as-synthesized Au NPs dispersed in water exhibit LSP resonance peaks at approximately 518 and 528 nm for diameters of 15 and 55 nm, respectively. Upon solvent exchange to ethanol, a slight red shift of 3 nm in the LSP resonance peaks is observed, attributable to ethanol’s higher refractive index relative to water.

Figure 2 presents the results of an initial screening to optimize the thickness of the top compact TiO_2_ sublayer in the double-layer architecture. The goal was to determine the minimum thickness required to fully encapsulate the Au NPs, thereby preventing carrier recombination at the NP surface.^53^ This thickness was also kept as low as possible to preserve potential plasmonic effects, which strongly depend on the distance to the active layer. In parallel, the influence of NP size was also investigated. All devices were fabricated with a fixed Au NP concentration of 0.15 wt %, corresponding to a single NP deposition cycle, and top sublayer thicknesses of 5, 10, and 15 nm, while maintaining a constant total compact TiO_2_ thickness of 35 nm. Such precise thickness control and conformality are only achievable with sputtering, as solution-processed TiO_2_ layers offer far less ability to define the encapsulation thickness with comparable accuracy. The PCE increases with the thickness of the top sublayer, with the highest values obtained for 15 nm TiO_2,_ surpassing the reference device without NPs by 1.0% (from 13.1 to 14.1%). For TiO_2_ thicknesses of 5 and 10 nm, Au NPs are not well encapsulated, likely resulting in recombination centers,^35^ which deteriorate device performance. For all tested thicknesses, devices incorporating 55 nm NPs consistently outperformed those with a diameter of 15 nm, indicating a clear advantage for the larger particle size. Consequently, the study focuses exclusively on 55 nm Au NPs from this point onward. Larger NP sizes (90 nm) were also evaluated; however, they could not be adequately encapsulated within the TiO_2_ layer, limiting their applicability.

**Figure 2:**
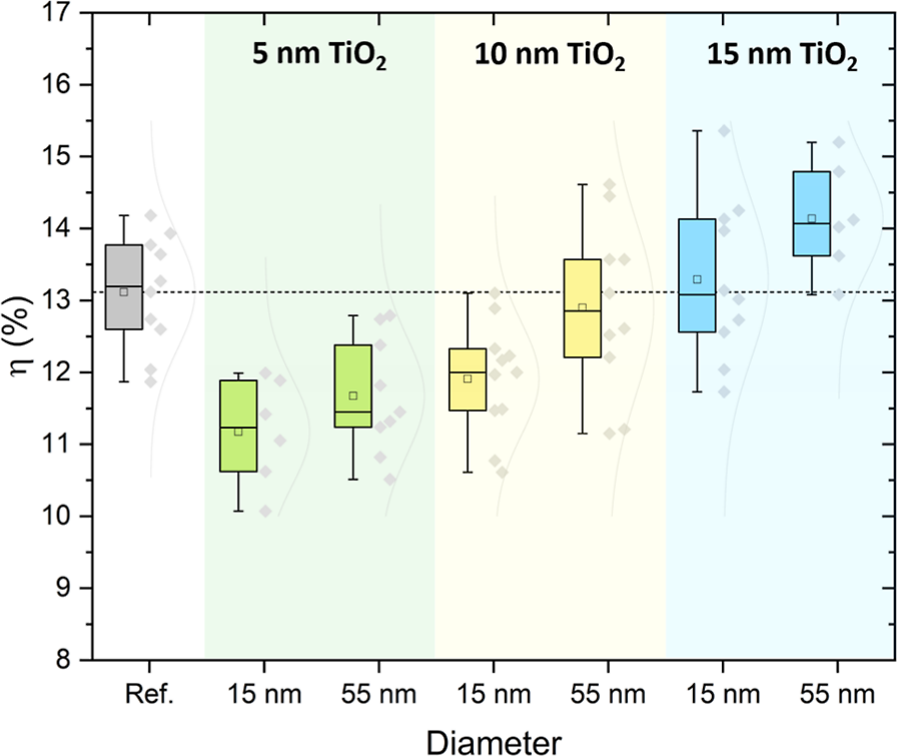
Statistical distribution of the PCE for different thicknesses (5, 10, 15 nm) of the top TiO_2_ sublayer, for devices incorporating Au NPs with diameters of 15 and 55 nm at a concentration of 0.15 wt % (measured under reverse-bias scanning). The thickness of the bottom TiO_2_ layer was adjusted to maintain a total TiO_2_ thickness of 35 nm. Between 6 and 11 samples were measured for each condition.

To investigate in greater detail the incorporation of 55 nm Au NPs, the effect of NP surface density was considered. For this purpose, 1, 2, 4, and 8 NP deposition cycles by spin-coating were carried out, corresponding to NP concentrations in the TiO_2_ layer of 0.15, 0.30, 0.60, and 1.20 wt %, respectively. Thicknesses of the top TiO_2_ sublayer of 15 and 20 nm were selected, guided by the encapsulation screening, which indicated 15 nm as the minimum top-TiO_2_ thickness delivering full NP encapsulation, while 20 nm was included to probe possible thickness-dependent effects. The surface of the double compact TiO_2_ layer containing Au NPs is shown in Figure 3a,b for NP concentrations of 0.15 and 1.20 wt %, respectively, for 15 nm top TiO_2_. The NP surface density was effectively controlled by the deposition method, being proportional to the number of deposition cycles. The images, acquired after a thermal annealing at 500 °C, reveal a uniform NP distribution and confirm that the NPs preserve their morphology upon annealing. The TiO_2_ layer exhibits a grain size of approximately 23 ± 6 nm, consistent with a nanocrystalline compact film.

**Figure 3:**
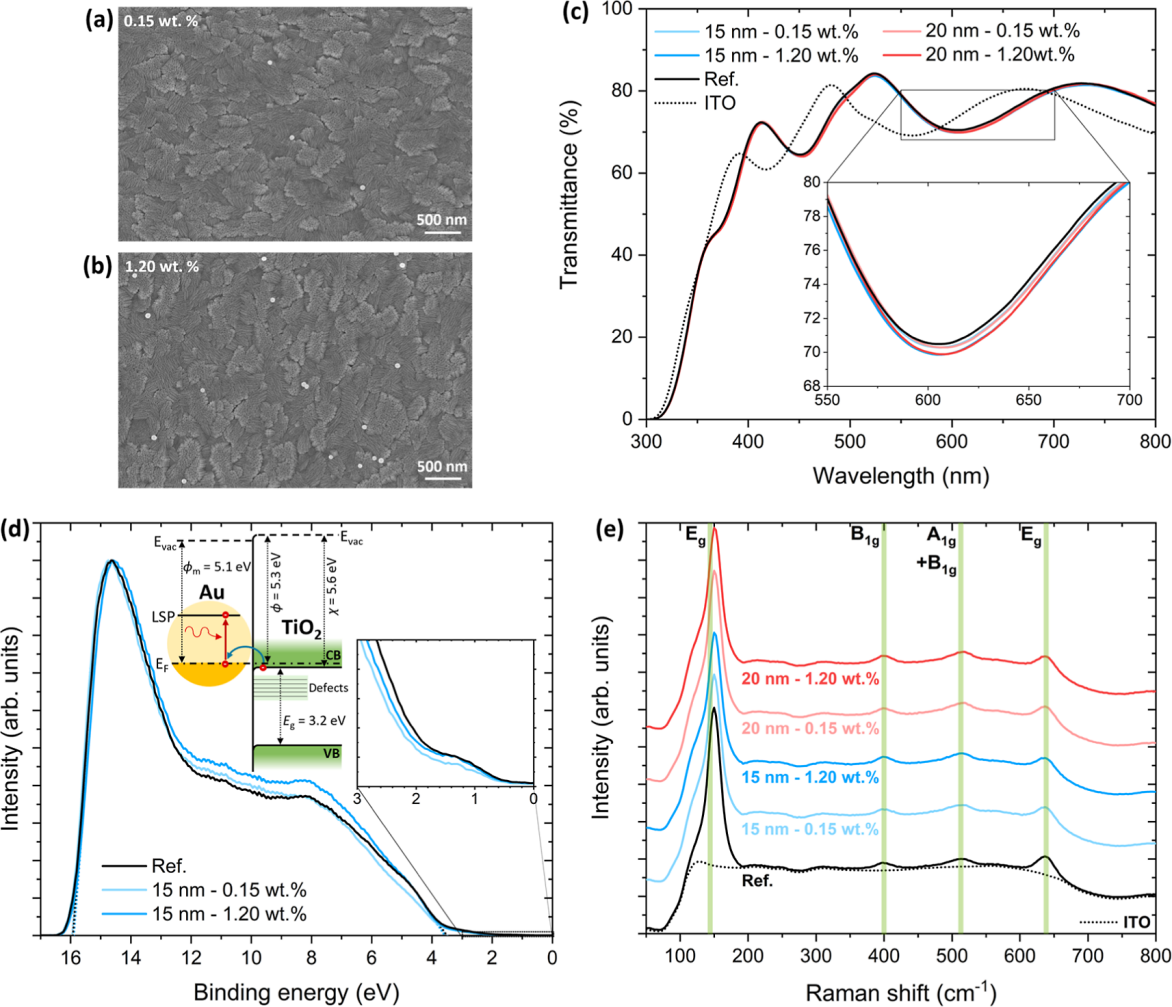
Top-view SEM image of the surface of the double compact TiO_2_ layer containing Au NPs for NP concentrations of (a) 0.15 wt % and (b) 1.20 wt %, for 15 nm top TiO_2_. (c) Transmittance spectra of the AuNPs@TiO_2_ films with top sublayer thicknesses of 15 and 20 nm, and NP concentrations of 0.15 and 1.20 wt %. (d) UPS spectra of AuNPs@TiO_2_ films with a top sublayer thickness of 15 nm, and NP concentrations of 0.15 and 1.20 wt %. Schematic energy band alignment between TiO_2_ and Au (inset). (e) Raman spectra of the AuNPs@TiO_2_ films with top sublayer thicknesses of 15 and 20 nm, and NP concentrations of 0.15 and 1.20 wt %.

Figure 3c shows the transmission spectra of the pristine TiO_2_ and AuNPs@TiO_2_ layers on ITO. The spectra reveal no significant differences between the reference TiO_2_ layer without NPs and the NP-containing structures. Nevertheless, a slight decrease in transmittance is observed around 600 nm for the samples with NPs, which can be attributed to light absorption by the LSP of Au NPs. The optical band gap of the TiO_2_ layers, with and without Au NPs, was estimated using the Tauc’s method (α*h*ν)^*n*^ = *A*(*h*ν – *E*_g_), where α is the absorption coefficient, *h*ν is the photon energy, *n* is the exponent variating with electronic transition, *A* is a proportionality constant, and *E*_g_ is the optical band gap (Figure S2). An indirect bandgap model (*n* = 1/2) was employed for the Tauc analysis. No significant difference was observed between pristine TiO_2_ and AuNPs@TiO_2_, with *E*_g_ being estimated at 3.17 eV at RT, indicating that the incorporation of Au NPs does not significantly modify the optical band gap of the TiO_2_. The obtained value is close to the expected band gap of anatase TiO_2_ and comparable with the values typically reported for TiO_2_ thin films (3.1–3.5 eV).^54–56^ The band gap was also evaluated by spectroscopic ellipsometry (Figure S3) using a Tauc-Lorentz model. Values close to 3.1 eV were obtained for the pristine TiO_2_ film and for samples with NP concentrations of 0.15 and 1.20 wt % (15 nm top TiO_2_ sublayer), in good agreement with UV–vis results. Ellipsometry further provided the film thicknesses, which were consistent with the nominal 35 nm: 36.7 nm for pristine TiO_2_, 37.7 nm for 0.15 wt %, and 38.4 nm for 1.20 wt %.

To investigate whether the incorporation of Au NPs affects the electronic structure of the ETL and the carrier extraction kinetics from the perovskite absorber, UPS measurements were performed on TiO_2_ films with and without NPs, using a top TiO_2_ sublayer thickness of 15 nm, as shown in Figure 3d. The secondary electron cutoff and valence band onset regions were extracted to determine the work function and the valence band maximum of each sample. The results indicate no significant difference between the pristine TiO_2_ films and the NP-containing films, with both exhibiting a work function of 5.3 eV and a valence band maximum at −8.8 eV (relative to vacuum level). Using the determined optical band gap, the conduction band minimum was estimated for both films and found to be essentially identical (−5.6 eV). These results demonstrate that the incorporation of Au NPs, even at a concentration of 1.20 wt %, does not measurably modify the electronic energy levels of the TiO_2_ layer. The obtained work function of 5.3 eV is consistent with values typically reported for compact anatase TiO_2_ films (∼4.5–5.5 eV),^57^ although it lies near the upper range of the reported values. The relative position of the Fermi level, apparently located slightly above the conduction band minimum, suggests the TiO_2_ films to be borderline or mildly degenerate, which may arise from the presence of oxygen vacancies or related donor-like defects, consistent with an n-type material.^58^ However, because UPS is a surface-sensitive technique, this degeneracy could primarily reflect near-surface downward band bending and electron accumulation,^57^ possibly enhanced by a higher density of oxygen vacancies near the surface, rather than a truly degenerate bulk TiO_2_. The corresponding energy band alignment between TiO_2_ and Au, derived from the UPS and UV–vis results, is shown in Figure 3d (inset). The downward band bending at the TiO_2_/Au interface suggests the formation of an electron accumulation layer on the interface; in contrast to the typical Schottky barrier formation in planar TiO_2_/Au contacts,^59^ no such barrier is expected here. Consistent with this alignment, no rectifying behavior is expected under our processing conditions; the Au/TiO_2_ interface displays ohmic behavior, enabling bidirectional electron transfer between the Au NPs and TiO_2_. This behavior can be attributed to the relatively high work function of the TiO_2_ film. The Fermi level alignment promotes a local accumulation of electrons within the near-surface region of TiO_2_, forming a shallow potential well where electrons are weakly confined and dynamically exchange with the surrounding TiO_2_ matrix. For completeness, Figure 3d also sketches the LSP excitation in the Au NPs; although LSPs do not correspond to discrete electronic levels, their excitation can generate hot carriers with energies above the Fermi level.

To further clarify the chemical environment and oxidation state of Ti within the TiO_2_ film, XPS measurements were performed on AuNPs@TiO_2_ layers with a 15 nm-thick top TiO_2_ sublayer, and the corresponding spectra are shown in Figure S4. In all samples, the Ti 2p spectrum exhibits a dominant doublet corresponding to Ti^4+^ at 458.6 eV (Ti 2p_3/2_) and 464.3 eV (Ti 2p_1/2_).^60^ A less intense doublet is also observed at lower binding energy values attributed to Ti^3+^ at 456.9 eV (Ti 2p_3/2_) and 462.7 eV (Ti 2p_1/2_), indicating the presence of oxygen vacancies (*V*_O_) in the TiO_2_ layer.^61^ These vacancies act as shallow donor centers, localizing electrons on neighboring Ti sites (Ti^3+^), while a small fraction becomes ionized and releases electrons to the conduction band, explaining the n-type behavior of TiO_2_ films.^61^ The Ti^3+^ component detected is consistent with the previous UPS data, where a shallow in-gap feature is observed at ∼1.0 eV below the Fermi level (0 eV), attributed to Ti 3d defect states associated with *V*_O_ in TiO_2_.^62^ Notably, the defect-band intensity is comparable for films with and without Au NPs, indicating that NP incorporation does not measurably perturb the electronic structure near the Fermi level. The presence of *V*_O_ in TiO_2_ is consistent with previous reports showing that sputtered TiO_2_ films contain significant defect densities, including Ti^3+^ species associated with oxygen vacancies and ion bombardment-induced lattice distortion,^48,49,63^ which limit interfacial selectivity and carrier transport.

From the XPS Ti 2p deconvolution, the fraction of reduced Ti was determined as Ti^3+^/(Ti^3+^+Ti^4+^) = 0.04 for samples with and without NPs. Assuming only Ti^3+^/Ti^4+^ species and charge neutrality in TiO_2‑δ_, the Ti^3+^ fraction is related to the oxygen deficiency according to Ti^3+^/(Ti^3+^ + Ti^4+^) = 2δ, yielding δ ≈ 0.02. This corresponds to a near-stoichiometric composition of approximately TiO_2-δ_ ≈TiO_1.98_ (O/Ti ≈1.98). Using the density of anatase TiO_2_ (ρ ≈3.9 g cm^–3^),^64^ the corresponding oxygen-vacancy density can be estimated as *N*_VO_ ≈5.9 × 10^20^ cm^–3^. This vacancy density falls within the range typically reported for slightly oxygen-deficient anatase TiO_2_ (10^19^–10^20^ cm^–3^), indicating a mildly reduced material.^65,66^ In the XPS O 1s spectra (Figure S4d–f), the main peak appears at 530.0 eV and is assigned to lattice oxygen bonded to Ti, while a secondary, less intense feature is observed at 531.5 eV. This minor component can be associated with surface carbon–oxygen species, surface hydroxyl groups, and oxygen atoms bonded to Ti^3+^ sites,^67^ consistent with the presence of *V*_O_ inferred from the Ti 2p spectrum. No detectable Au 4f peaks were observed in any of the samples, confirming that the Au NPs are effectively encapsulated within the TiO_2_ layer and are not exposed to the surface.

To further assess potential change in the crystalline structure after Au NPs incorporation, Raman spectroscopy was performed on pristine TiO_2_ and AuNPs@TiO_2_ films with top sublayer thicknesses of 15 and 20 nm, as shown in Figure 3e. No appreciable differences are observed in the spectra of the various samples, further confirming that the presence of Au NPs does not significantly affect the crystalline properties of the TiO_2_ layer. For all spectra, however, the anatase *E*_g_ mode appears at ∼150 cm^–1^, blue-shifted by 6 cm^–1^ from its nominal position at 144 cm^–1^, and displays a significant line width (full width at half maximum (FWHM) ∼23 cm^–1^).^68^ Such a blue shift and broadening are consistent with the presence of *V*_O_, as supported by XPS and UPS analyses. Oxygen deficiency in TiO_2_ reduces the O/Ti ratio, inducing local lattice distortions and microstructural inhomogeneities that harden the *E*_g_ phonon mode and shift it toward higher wavenumbers.^69^

To gain further insight into the structural properties of the TiO_2_ films, XRD measurements were performed on the same samples (Figure S5). Due to the relatively thin film thickness, only weak diffraction peaks corresponding to the (101), (004), and (200) planes of anatase TiO_2_ were detected, located at 2θ ≈ 25.31°, 38.13°, and 48.08°, respectively. The (200) reflection shows no measurable shift compared with the standard position (48.09°, PDF#00-021-1272), whereas the (004) peak is shifted by ∼ 0.31° from its nominal position (37.82°), suggesting a slight deformation along the [001] direction. Using the (004) shift, we obtain *c* = 9.433 Å (versus *c*_0_ = 9.507 Å), i.e. an out-of-plane strain ε_*c*_ ≈−0.78%. To rationalize the blue shift of the anatase *E*_g_ mode in the Raman results, the lattice distortion was treated as dominated by the volumetric component of strain. As the (200) peak does not shift, ε_*a*_ ≈0, hence volumetric strain Δ*V*/*V* ∼ε_*c*_. The associated equivalent hydrostatic pressure *P*_eq_ = −*K*ε_*c*_ is ∼1.4–1.5 GPa, with *K* ∼179–189 GPa for anatase.^70^ Using the piezospectroscopic coefficient for the *E*_g_ mode, dω/d*P* ∼2–3 cm^–1^ GPa^–1^,^71,72^ the expected Raman shift of *E*_g_ mode due to strain is ∼3–5 cm^–1^, in good agreement with the experimentally observed ∼6 cm^–1^ blue shift.

Figure 4a presents the statistical distribution of PCE values for PSCs fabricated with pristine TiO_2_ and TiO_2_ containing 55 nm Au NPs at concentrations ranging from 0.15 wt % to 1.20 wt %, for top TiO_2_ sublayer thicknesses of 15 and 20 nm. An enhancement in PCE is observed at the lowest NP concentration (0.15 wt %), with the average PCE increasing from 12.9 to 14.2% for 15 nm, and to 13.2% for 20 nm TiO_2_. The relative improvement is more pronounced for the thinner top sublayer. As the NP concentration increases, the PCE gradually decreases, and for concentrations above 0.30 wt % the average PCE falls below that of the reference device without NPs. Figure S6 summarizes the statistical distribution of the other photovoltaic parameters, namely short-circuit current density (*J*_sc_), open-circuit voltage (*V*_oc_), and fill factor (FF). The *J*_sc_ follows the same trend as the PCE, with an improvement over the reference for low NP concentrations, more pronounced for a 15 nm TiO_2_ top sublayer. The highest increase is from 18.4 mA cm^–2^ to 19.8 mA cm^–2^ for 0.15 wt % and 15 nm TiO_2_. A slight improvement is also observed in *V*_oc_ at low NP concentrations, with a maximum increase from 1.052 to 1.063 V for 0.15 wt % and 20 nm TiO_2_. In contrast, the FF decreases with the incorporation of NPs and further decreases as the NP concentration increases. For 1.20 wt % and 15 nm TiO_2_, the FF drops from 66% to 61%. The variation in PCE is mainly driven by changes in *J*_sc_ and FF, whereas the variations in *V*_oc_ are small, as observed in the *J*–*V* curves shown in Figure 4b,c. The origin of this efficiency behavior is further investigated and discussed below. Photovoltaic parameters of the devices with pristine TiO_2_ and AuNPs@TiO_2_ for 0.15 and 1.20 wt % NP concentration are listed in Table 1.

**Figure 4:**
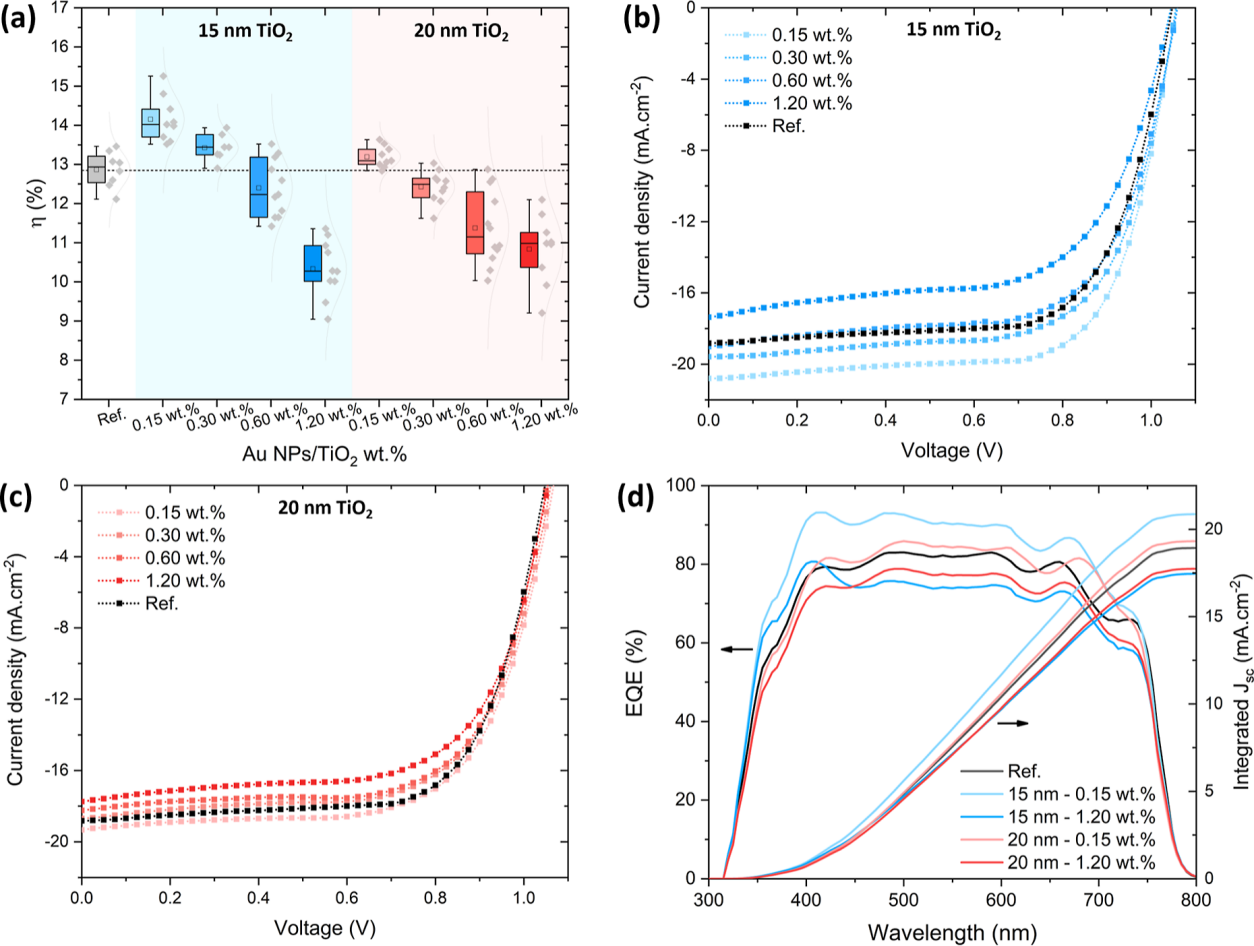
(a) Statistical distribution of the PCE for different thicknesses (15, 20 nm) of the top TiO_2_ sublayer, for devices incorporating 55 nm Au NPs at concentrations of 0.15, 0.30, 0.60, and 1.20 wt % (measured under reverse-bias scanning). Between 7 and 12 samples were measured for each condition. *J*–*V* curves under 1 sun illumination of PSCs with TiO_2_ and AuNPs@TiO_2_ for different NP concentrations for thicknesses of (b) 15 nm and (c) 20 nm of the top TiO_2_ sublayer. (d) EQE spectra for the devices based on TiO_2_ and AuNPs@TiO_2_.

**Table 1:** Photovoltaic Parameters (Average ±Standard Deviation) of the Devices With Pristine TiO_2_ and AuNPs@TiO_2_

	*J*_sc_ (mA cm^–2^)		*V*_oc_ (V)		FF (%)		PCE (%)	
devices	average	best	average	best	average	best	average	best
ref.	18.4 ± 0.4	18.90	1.052 ± 0.004	1.0599	66 ± 1	68.6	12.9 ± 0.5	13.46
15 nm—0.15 wt %	19.8 ± 0.6	20.82	1.056 ± 0.004	1.0606	67 ± 2	69.7	14.2 ± 0.6	15.26
15 nm—1.20 wt %	16.5 ± 0.6	17.42	1.041 ± 0.005	1.0498	61 ± 1	62.5	10.3 ± 0.7	11.36
20 nm—0.15 wt %	18.8 ± 0.3	19.33	1.063 ± 0.004	1.0692	66 ± 1	68.0	13.2 ± 0.3	13.64
20 nm—1.20 wt %	16.5 ± 0.8	17.75	1.050 ± 0.006	1.0570	62 ± 2	65.2	10.8 ± 0.9	12.10

Figure 4d displays the EQE spectra of devices with and without Au NPs, normalized to the *J*_sc_ values obtained from the *J*–*V* curves. The observed variations in EQE follow a nearly uniform trend over the entire spectral range, with no selective enhancement near the LSP resonance wavelength of the Au NPs. This indicates that the increase in *J*_sc_ observed for low NP concentrations is not primarily due to enhanced light absorption in the perovskite layer via near-field effects or scattering-induced optical path length extension. This conclusion is further supported by the absorption spectra of the devices (Figure S7), without the Au back contact, which show negligible differences between samples with and without NPs. These results suggest that performance enhancement is primarily governed by electronic processes rather than plasmonic light-harvesting effects.

The previous interpretation is corroborated by finite-difference time-domain (FDTD) optical simulations (Figure S8) spanning NP diameters of 10–200 nm and interspacing values from 30–1000 nm (square lattice). Consistently, simulations show no optical gain for any NP size/interspacing combination. At smaller interspacings the response becomes loss-dominated due to parasitic absorption in Au NPs, leading to a substantial reduction in *J*_sc_. For the experimentally used densities, the model predicts no substantial optical losses: the calculated *J*_sc_ for the NP-free reference is 21.47 mA cm^–2^, and for 1.20 wt % (∼1000 nm interspacing), the simulated *J*_sc_ loss remains <0.1 mA cm^–2^ relative to the reference.

XRD patterns (Figure S9) for perovskite films grown on TiO_2_ with and without Au NPs exhibit comparable peak positions and full widths at half-maximum, indicating no measurable change in average crystallinity. Likewise, top-view SEM (Figure S10) reveals similar grain-size distributions, confirming that NP incorporation in the ETL does not affect the bulk morphology of the absorber. Thus, any additional roughness introduced by the embedded NPs in the top TiO_2_ sublayer (15 or 20 nm) does not propagate into the perovskite in a way that modifies its bulk properties. Local interfacial variations, such as strain, defect density, or facet-selective nucleation, may nevertheless arise at the TiO_2_/perovskite interface, where growth is sensitive to surface roughness.^73,74^ Such results indicate that NP-induced modifications are primarily interfacial rather than in bulk.

As a preliminary observation, the photovoltaic parameters do not indicate an improvement in TiO_2_ transport layer conductivity upon Au NP incorporation. Across NP concentrations, the FF value does not increase and, at higher NP concentration, even decreases, a behavior inconsistent with a reduction of transport resistance in the ETL.^75^ This suggests that the dominant changes must be in interface selectivity and recombination.

To further elucidate these effects, electrochemical impedance spectroscopy (EIS) measurements were performed on PSCs with pristine TiO_2_ and AuNPs@TiO_2_ ETLs to investigate the impact of NP incorporation on the interface charge recombination dynamics. Figure 5a shows the Nyquist plots measured in the dark at 0.75 V for devices with pristine TiO_2_ and AuNPs@TiO_2_ at 0.15 and 1.20 wt % NP concentration, using top TiO_2_ sublayers of 15 and 20 nm. Under this forward bias, the Nyquist semicircle is dominated by recombination in the injected-diode regime.^76,77^ The data were fitted with an equivalent circuit comprising a series resistance (*R*_s_) in series with a recombination branch, where a recombination resistance (*R*_rec_) is in parallel with a constant phase angle element (CPE), which represents a nonideal capacitance.^78^ Here, *R*_s_ accounts for contributions from external wiring, contact resistances, and the ITO sheet resistance. Under these conditions, transport contributions are negligible in the observed semicircle, as the transport branch appears at much higher frequencies and remains masked near *R*_s_.^31^ Incorporation of Au NPs into TiO_2_ increases *R*_rec_ under all tested conditions (Table 2). This behavior is consistent with interface defect passivation at the TiO_2_/perovskite interface, thereby reducing interface recombination losses,^79,80^ and with a more selective ETL that enhances minority-carrier blocking under forward bias.^81^ Both effects reduce d*J*_dark_/d*V*, inversely proportional to *R*_rec_, reflecting a reduced recombination rate. The increase in *R*_rec_, thus, may account for the observed *J*_sc_ enhancement for 0.15 wt % NP concentration. Although passivation effects typically result in a *V*_oc_ increase by reducing interface recombination,^82^ in this case, *V*_oc_ remains nearly unchanged, suggesting that other concurrent processes may offset the expected *V*_oc_ gain. The more pronounced rise in *R*_rec_ at 1.20 wt % than at 0.15 wt % may indicate an enhanced interface passivation and a more effective suppression of interface recombination at higher NP densities. No significant difference in *R*_rec_ is observed between devices with 15 and 20 nm top TiO_2_ sublayers, suggesting that the effect of NP concentration dominates over TiO_2_ thickness-related variations. The effective recombination capacitance (*C*_rec_), calculated from the CPE of the recombination branch, decreases across all NP-containing devices, consistent with interface passivation by the Au NPs, which lowers the density of interfacial states and thereby reduces charge accumulation and recombination.^78^

**Figure 5:**
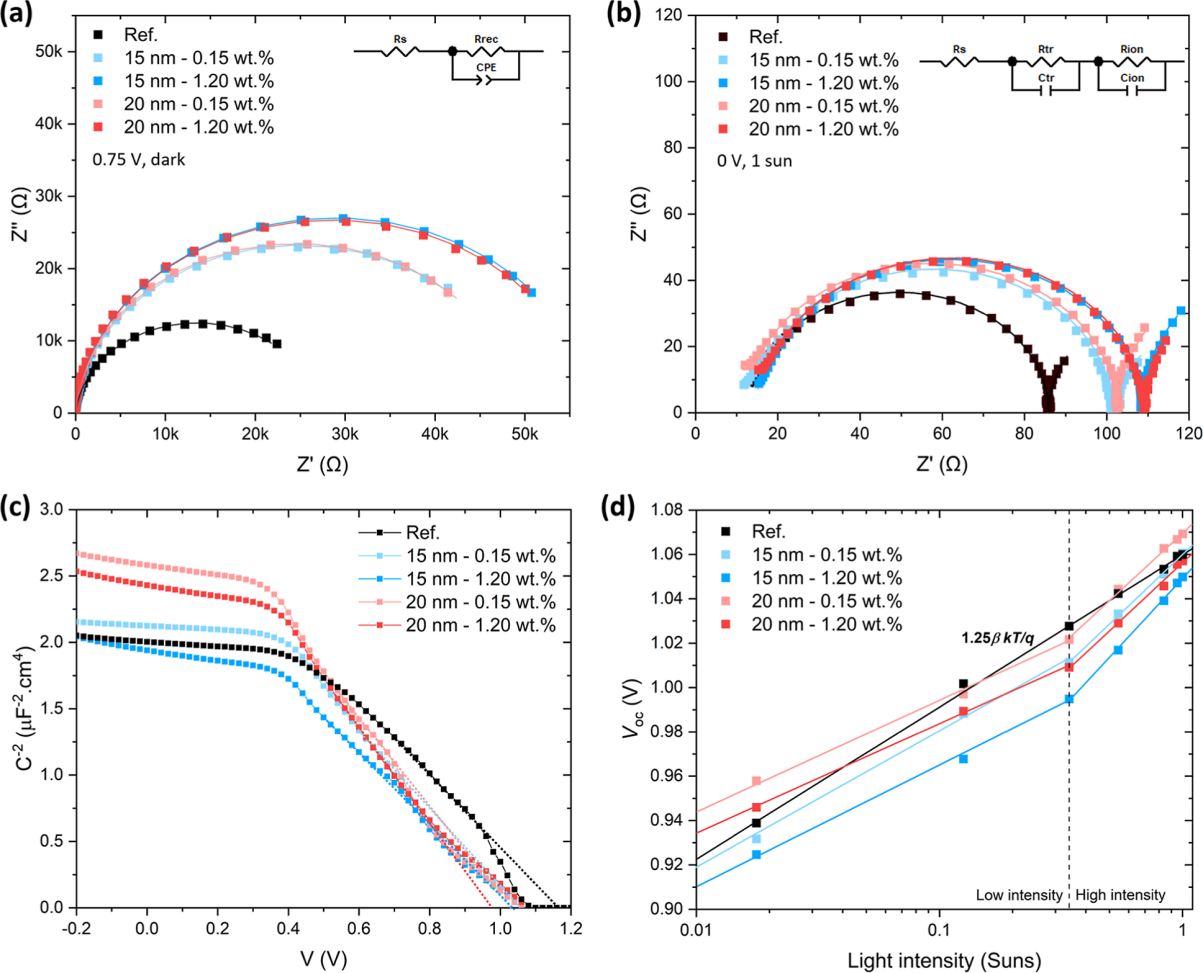
Nyquist plots of PSCs with TiO_2_ and AuNPs@TiO_2_ ETLs, from EIS measurements under (a) 0.75 V in the dark and (b) 0 V under 1 sun illumination. The fittings shown were obtained using the equivalent circuit model displayed in the inset. (c) Mott–Schottky plots recorded at 10 kHz in the dark. (d) *V*_oc_ as a function of light intensity for the same devices.

**Table 2:** Electrical Output Characteristics by EIS of the Devices with Pristine TiO_2_ and AuNPs@TiO_2_

	0.75 V, dark			0 V, 1 sun					
devices	*R*_s_ (Ω)	*R*_rec_ (kΩ)	*C*_rec_ (nF)	*R*_s_ (Ω)	*R*_tr_ (Ω)	*C*_tr_ (nF)	*V*_bi_ (V)	*N* (×10^17^ cm^–3^)	*n*_high*I*_/*n*_low*I*_
ref.	9.8	27.1	21.4	13.1	72.8	10.2	1.16	7.65	1.25
15 nm—0.15 wt %	10.9	49.5	15.3	14.6	86.8	10.8	1.06	7.30	1.83/1.11
15 nm—1.20 wt %	15.9	57.6	15.9	15.6	92.7	9.5	1.03	7.93	2.36/1.09
20 nm—0.15 wt %	11.8	49.2	15.5	12.2	90.3	6.1	1.03	6.40	2.00/1.01
20 nm—1.20 wt %	15.7	57.0	14.7	15.6	93.5	6.4	0.98	5.90	2.09/1.02

To elucidate why 1.20 wt % yields lower *J*_sc_, *V*_oc_, and FF despite stronger passivation than 0.15 wt %, transport-related losses were isolated via an EIS measurement under 1 sun at 0 V (Figure 5b). Under these conditions, the response is expected to be dominated by a high-frequency (HF) semicircle that reports an effective charge-transfer resistance (*R*_tr_) across the ETL-perovskite interfaces plus conduction through the transport layers.^83^ *R*_tr_ increases in Au NPs@TiO_2_ devices and rises with NP concentration (Table 2). This behavior is consistent with a transport constriction effect that limits charge transport through the ETL. Embedded Au NPs behave as electrically isolated metallic islands that transiently store electrons close to their surface, acting as a floating gate.^84^ According to the band alignment estimated from UPS (Figure 3d), charge accumulation in the TiO_2_/Au NPs interface is energetically favorable, consistent with their role as electron sinks.^44,85,86^ The stored charge electrostatically depletes the surrounding TiO_2_,^87,88^ which modulates the current pathways and forces carriers to detour around depleted regions, increasing transport resistance. This rise in *R*_tr_ explains the FF loss and, at higher NP concentration, the reduction in *J*_sc_. At the same time, the accumulation of free electrons at the NP surface leaves nearby oxygen-vacancy sites positively charged, creating an internal electric field that repels holes from the TiO_2_/perovskite interface. This additional field would strengthen the hole-blocking character of the ETL and further suppress interface recombination, which may explain the origin of the enhanced ETL selectivity previously inferred from EIS. A reduction in the fitted HF capacitance (*C*_tr_) is observed for devices with a 20 nm TiO_2_ top sublayer upon the introduction of Au NPs into the ETL. This decrease is consistent with the electron-sink behavior of the Au NPs, which introduces an additional series capacitance, thereby lowering the measured capacitance.^78,89^ When the top TiO_2_ sublayer is 15 nm, the Au NPs lie closer to the TiO_2_/perovskite interface. As a result, the NP-induced captured charge couples predominantly in parallel with interface capacitances, making the electron-sink signature less effective in the HF capacitance. In addition to the transport-dominated HF arc, a second lower-frequency semicircle is resolved, commonly associated with ionic interface polarization processes.^78^ For this low-frequency arc, the fitted *R*_p_–*C*_p_ branch values are not included in Table 2, as the associated uncertainty was considerable to yield reliable values. An increase in *R*_s_ is observed upon Au NP incorporation, more pronounced at 1.20 wt %. This behavior is consistent with charge accumulation at the Au NPs, which depletes the surrounding TiO_2_ and raises the effective resistivity of the ETL. The resulting deterioration appears partly as an ohmic, frequency-independent contribution to *R*_s_, and partly as an interfacial and kinetic contribution manifested in *R*_tr_. In summary, low NP densities (0.15 wt %) primarily improve selectivity and defect passivation, establishing an alternative, nonplasmonic route for performance enhancement, while higher densities (1.20 wt %) introduce transport bottlenecks due to electron sinks, which limit device performance. This behavior differs from several previous studies in which metallic NPs were reported to enhance charge transport by lowering *R*_tr_ through increased ETL conductivity or hot-electron injection,^28,35,40^ as well as to facilitate carrier extraction across the ETL/perovskite interface.^31^

Figure 5c presents the Mott–Schottky analysis of the devices. In such analysis, the capacitance associated with the depletion region is expected to follow the relation 1/*C* ^2^ = 2­(*V*_bi_ – *V*)/*q*εε_0_ *N*,^90^ where *V*_bi_ is the built-in potential, *q* is the elementary charge, ε is the relative dielectric constant of the perovskite absorber, calculated based on the geometric capacitance, ε_0_ is the vacuum permittivity and *N* is the density of fixed charge forming the depletion region. A systematic decrease of the apparent *V*_bi_ is observed upon Au NP incorporation (Table 2), consistent with an electron-sink effect in the ETL. The accumulated charge at the embedded NPs introduces an additional series capacitance that partially screens the internal electric field. This effect is apparent and does not necessarily correspond to a genuine reduction in the device *V*_bi_.^89^ For higher NP densities, *V*_bi_ decreases further, reflecting the more substantial sink effect. At the same time, *N* decreases for 20 nm TiO_2_, which is attributed to the influence of the series capacitance that increases the module of the 1/*C* ^2^–*V* slope, rather than to an actual change in the fixed charge density of perovskite. This behavior is consistent with the Nyquist-derived values of *C*_tr_, which only show a reduction for 20 nm TiO_2_. Importantly, the reduction in the *V*_bi_ provides a consistent explanation for why the *V*_oc_ improvement with Au NPs remains modest (maximum increase from 1.052 to 1.063 V) despite the significant increase in *R*_rec_. The interfacial passivation effect that tends to raise *V*_oc_ is partially counteracted by the electric-field screening associated with the electron-sink behavior of the embedded Au NPs. The combined action of these opposing effects results in a *V*_oc_ that changes only marginally. While the Mott–Schottky analysis provides a quantitative measure of *V*_bi_, a direct quantification of the localized charge stored at the Au NPs and its impact on *V*_oc_ would require operando measurements of the interfacial potential (e.g., Kelvin probe–based techniques). Such measurements are particularly challenging to perform on complete devices under simultaneous illumination and electrical bias and are therefore beyond the scope of the present study.

To probe recombination mechanisms under photovoltaic operation, light intensity-dependent *J*–*V* measurements were performed, and *V*_oc_ was plotted against ln­(*I*) (Figure 5d). In this representation, the slope directly yields the ideality factor (*n*) according to d*V*_oc_/d ln­(*I*) = *n*β*kT*/*q*, where β = *d*ln­(*J*_sc_)/*d*ln­(*I*) accounts for the dependence *J*_sc_ ∝ *I* ^β^ (with β determined for each device; Figure S11), *k* is the Boltzmann constant, and *T* is the temperature.^91^ This analysis probes recombination under illumination and open-circuit conditions, in contrast to the EIS measurements at 0.75 V in the dark, which revealed enhanced interface passivation and selectivity upon Au NP incorporation into the ETL. The *V*_oc_–ln­(*I*) curve of the reference device with pristine TiO_2_ exhibits a linear behavior with an ideality factor of 1.25 (Table 2). This value is consistent with predominantly bimolecular recombination with a minor contribution from Shockley-Read-Hall (SRH) processes.^92^ In contrast, all NP-containing devices display two distinct regimes depending on light intensity. At high intensities (above 0.3 suns), the ideality factor increases to 1.83 and 2.36 for 0.15 and 1.20 wt % NP concentration (15 nm TiO_2_), respectively, evidencing a transition to SRH-dominated recombination. At low intensities, *n* approaches unity (1.11 for 0.15 wt % and 1.09 for 1.20 wt %, for 15 nm TiO_2_), suggesting that the Au NPs capture a significant fraction of the photogenerated charge, thereby suppressing recombination near the ETL/perovskite interface. At higher intensities, however, the higher carrier generation rate permanently saturates the NPs with stored charge. Under open-circuit conditions, where no charge extraction occurs, this sink effect induces a depletion region in TiO_2_ that forces carriers to remain longer near the ETL/perovskite interface, thereby enhancing SRH recombination.

Steady-state PL and TRPL (Figure 6) measurements were performed with excitation from the ETL side. Under this geometry, the detected emission predominantly originates from perovskite regions close to the TiO_2_/perovskite interface, making the measurement particularly sensitive to interface recombination and carrier extraction kinetics. Steady-state PL spectra for films on pristine TiO_2_ and AuNPs@TiO_2_ show no discernible spectral shift of the emission maximum, consistent with an unchanged bulk optoelectronic band gap. The overall PL intensity increases with the incorporation of Au NPs, consistent with interface passivation, which suppresses nonradiative recombination. This effect also explains the higher PL intensity observed for thicker (20 nm) TiO_2_ layers at higher NP concentrations. On the other hand, for thinner (15 nm) TiO_2_ layers, a smaller PL enhancement is observed as the Au NP concentration increases from 0.15 to 1.20 wt %. This can be attributed to the electron-sink effect at high NP concentrations, which partially screens the local drift field near the TiO_2_/perovskite interface. As a result, carriers tend to remain near the ETL/perovskite interface, thereby enhancing SRH nonradiative recombination and mitigating the PL enhancement, despite the improved passivation expected at higher NP concentrations. This behavior is particularly pronounced for 15 nm TiO_2_, consistent with the larger ideality factor increase observed under high illumination intensity in the *V*_oc_–ln­(*I*) analysis, due to the closer proximity of the NPs to the perovskite interface.

**Figure 6:**
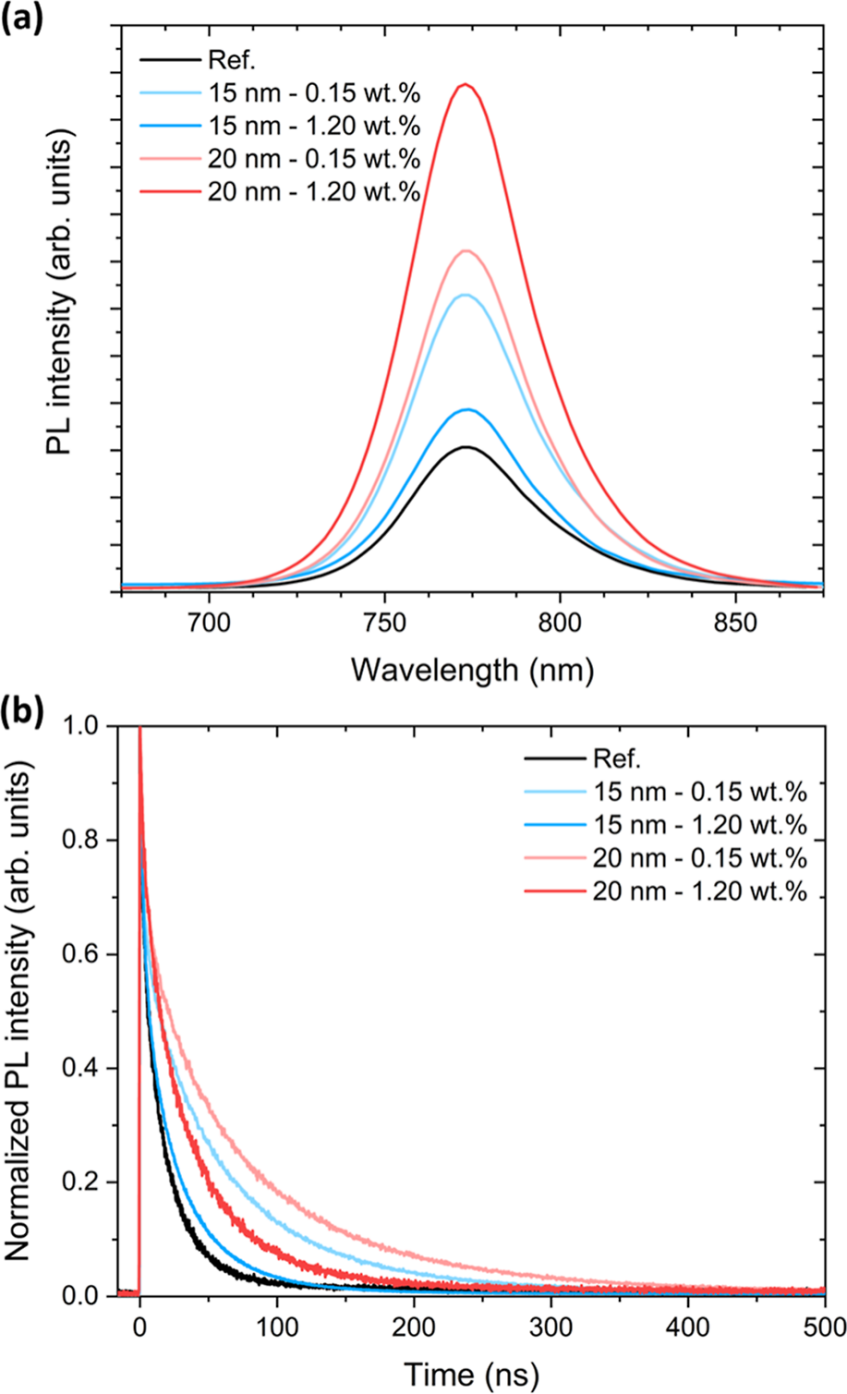
(a) Steady-state PL spectra and (b) normalized TRPL of the perovskite in devices with pristine TiO_2_ and AuNPs@TiO_2_ for different thicknesses (15, 20 nm) of the top TiO_2_ sublayer, for devices incorporating 55 nm Au NPs at concentrations of 0.15 and 1.20 wt %.

TRPL transients (Figure 6b) were acquired at the maximum PL peak and fitted with biexponential decay fits (Table 3). The fast component of the PL lifetime (τ_fast_), commonly assigned to interface recombination and carrier extraction at the ETL/perovskite interface,^93^ increases from 2.5 ns (pristine TiO_2_) to 3.3 ns (0.15 wt %) and 3.5 ns (1.20 wt %) for 15 nm TiO_2_. A similar trend is observed for 20 nm TiO_2_. This finding again supports the presence of interface defect passivation, in line with the higher *R*_rec_ observed in EIS. This behavior contrasts with several previous reports, where the incorporation of metal NPs led to shorter PL lifetimes due to accelerated charge extraction from the perovskite absorber.^28,31,33^ The slow component (τ_slow_), often associated with radiative recombination and detrapping from shallow states,^94^ is 21.5 ns for the reference device. This value is, in fact, limited by nonradiative recombination and by carrier extraction processes in a complete ETL/perovskite/HTL device.^95^ The built-in drift field promotes the separation of photogenerated electron–hole pairs, further influencing the effective PL lifetime.^96^ Upon Au NP incorporation, τ_slow_ also increases for both concentrations, but maximizes at 0.15 wt % NP density (63.0 ns for 0.15 wt %, and 31.9 ns for 1.20 wt %, for 15 nm TiO_2_). This enhancement of τ_slow_ reflects the reduced interfacial losses, following the transport of photogenerated electrons toward the TiO_2_/perovskite interface. At higher NP concentrations, the electron-sink and depletion effect partially screen the local drift field near the TiO_2_/perovskite interface. Consequently, carrier extraction becomes less efficient, and carriers tend to remain near the ETL/perovskite interface. In this region, SRH nonradiative recombination is more probable, tempering the τ_slow_ enhancement, despite the continued interface passivation evidenced by τ_fast_.

**Table 3:** Biexponential Decay Dynamics by TRPL of the Devices with Pristine TiO_2_ and AuNPs@TiO_2_

devices	τ_fast_ (ns)	τ_slow_ (ns)
ref.	2.5	21.5
15 nm—0.15 wt %	3.3	63.0
15 nm—1.20 wt %	3.5	31.9
20 nm—0.15 wt %	5.8	80.3
20 nm—1.20 wt %	7.5	43.9

To better understand how Au NP incorporation affects the intrinsic conductivity of TiO_2_, vertical ITO/TiO_2_/Ag *J*–*V* measurements were performed (Figure 7a–c). These reveal identical dark conductivity for pristine TiO_2_ and AuNPs@TiO_2_, indicating that Au NP incorporation does not introduce any additional large-scale transport barrier. This further rules out a resistive transport effect at the Au/TiO_2_/Au interfaces as the predominant mechanism responsible for the observed increase in *R*_tr_. Instead, the dominant effect of Au NP incorporation is charge capture and transport constriction induced by depletion rather than current blocking, which becomes relevant only at low current densities. The current densities in the ITO/TiO_2_/Ag structures are orders of magnitude larger than the photocurrents in PSC operation, so any field-effect modulation by the electron sink mechanism becomes saturated and is not expected to produce a measurable drop in the macroscopic conductivity of TiO_2_ with NPs. Under 1 sun, the pristine TiO_2_ sample exhibits no immediate change in conductivity upon switching on the light; however, after 10 min of continuous exposure, the conductivity increases measurably. This conductivity gain corresponds to a persistent photoconductivity,^18^ where photogenerated charges passivate defects in the TiO_2_. Indeed, at an applied bias of 0.6 V, the current density increases from 200 mA cm^–2^ in the dark to 235 mA cm^–2^ after 10 min of light soaking. Such a significant increase cannot be explained by the instantaneous photocarrier population, confirming that defect passivation is the dominant origin. The effect disappears when a UV-blocking filter is used, demonstrating that only photons with energy above the TiO_2_ band gap contribute to the photoconductivity enhancement, while sub-bandgap excitation has no impact. In contrast, AuNPs@TiO_2_ samples show no change in conductivity even after 10 min of illumination. This behavior indicates that electron-sink charging at the Au NPs prevents photogenerated carriers from passivating TiO_2_ defect states.

**Figure 7:**
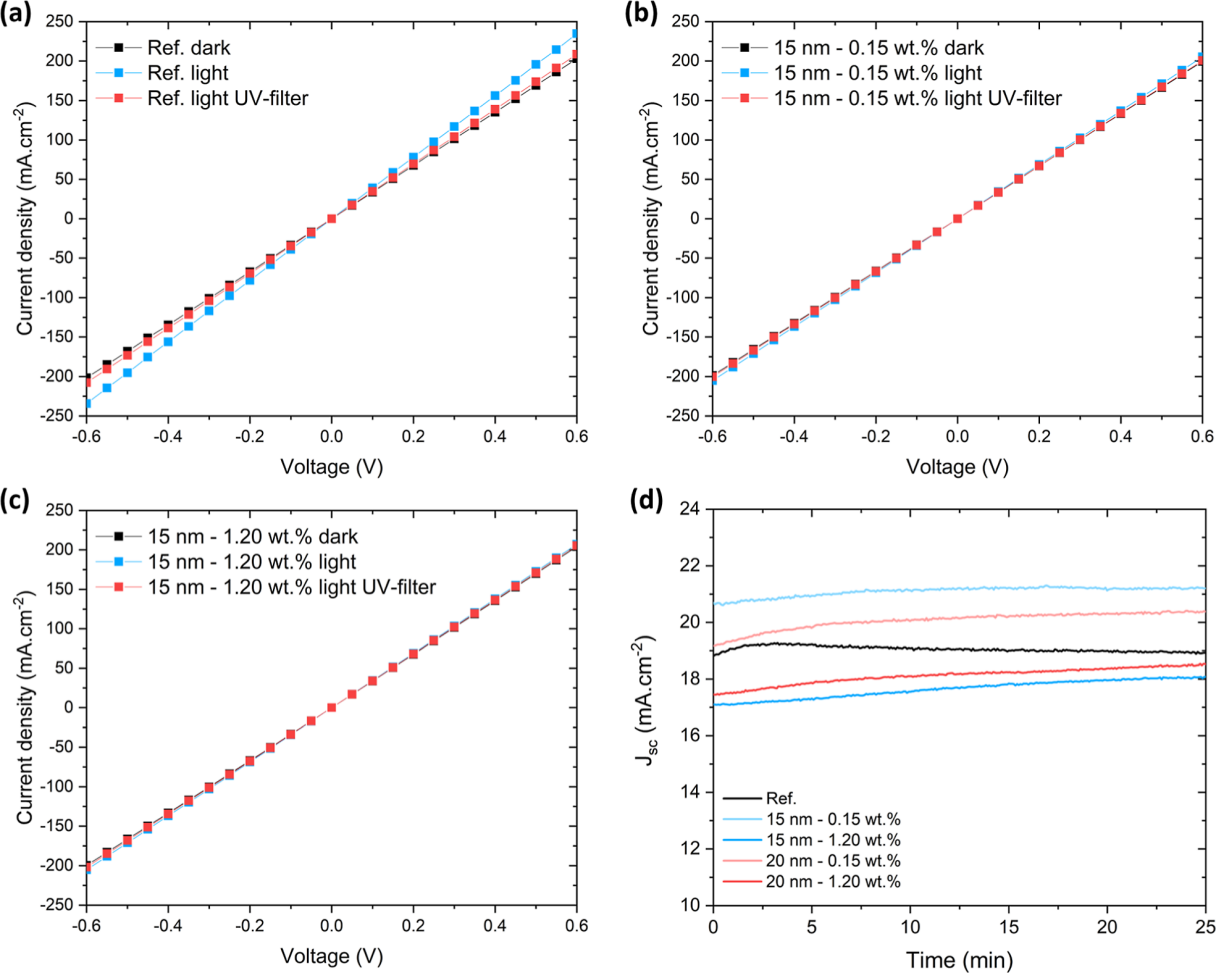
*J*–*V* curves of the vertical conductivity for (a) pristine TiO_2_ and AuNPs@TiO_2_ with (b) 0.15 wt % and (c) 1.20 wt % Au NP concentration, using 15 nm TiO_2_, in an ITO/ETL/Ag structure, measured under dark and 1 sun illumination (with and without a 400 nm UV filter). (d) Time evolution of *J*_sc_ for devices with TiO_2_ and AuNPs@TiO_2_ under continuous 1 sun illumination.

Another relevant observation, following the photocurrent measurements, is the time evolution of the *J*_sc_ under continuous 1 sun illumination (Figure 7d). For the pristine TiO_2_ device, *J*_sc_ exhibits an initial rise during the first few minutes, followed by a gradual decay over time. The initial increase is consistent with the UV-activated persistent photoconductivity of TiO_2_, which enhances the ETL conductivity. The subsequent decrease is attributed to photoinduced interfacial degradation at the TiO_2_/perovskite interface, driven by the photocatalytic activity of TiO_2_ under UV illumination,^97^ which progressively increases interface recombination losses. Although most photogenerated carriers in TiO_2_ contribute to the photocurrent under short-circuit conditions, a small fraction can still participate in surface reactions at the illuminated TiO_2_/perovskite interface, particularly under UV excitation. Electrons photogenerated in the TiO_2_ conduction band can reduce adsorbed molecular oxygen at Ti^3+^ sites, forming reactive oxygen species such as superoxide, which initiate oxidative reactions that degrade the perovskite, e.g., deprotonation of the methylammonium cation and promoting formation of PbI_2_.^98^ Photogenerated holes in the TiO_2_ valence band can also oxidize surface species such as halide anions, further promoting radical-mediated degradation cascades that damage the perovskite layer.^99^ In contrast, devices incorporating Au NPs exhibit a continuous, monotonic increase in *J*_sc_ throughout the entire 25 min measurement window. This behavior reflects the electron-sink function of the embedded Au NPs, which suppresses the persistent photoconductivity of TiO_2_ by capturing photogenerated electrons and mitigating UV-driven degradation at the ETL/perovskite interface. The slow increase in *J*_sc_ under 1 sun in the Au NP-based devices is most plausibly attributed to trap filling within the perovskite layer during light soaking.^100,101^ It should be emphasized that this *J*_sc_ time-evolution analysis reported here provides exclusively a short-term mechanistic diagnostic of the TiO_2_/perovskite interfacial behavior, rather than a measure of long-term operational stability. Such short-term photocurrent and light-soaking measurements are widely employed in the literature as sensitive probes of interfacial charge dynamics and light-induced effects in PSCs.^31,102^ A meaningful assessment of long-term device stability would require fully encapsulated devices and ISOS-compliant aging protocols, which are beyond the scope of the present work.

Although the detailed analysis in this work focused on the 55 nm Au NPs, the superior performance of these larger NPs compared with the 15 nm ones, observed consistently in the initial screening (Figure 2), can be rationalized considering the electronic mechanisms identified for the optimized architecture. In particular, the larger absolute metal/oxide interfacial area of the 55 nm NPs is expected to enhance their charge-storage capability, giving rise to more extended depletion regions in the surrounding TiO_2_ and thereby improving interfacial selectivity. At the same time, the smaller NPs possess a higher surface-to-volume ratio, which increases the fraction of reactive facets that may contribute to interfacial recombination. While complementary measurements would be needed to fully quantify these effects for the 15 nm NPs, the trends observed across the device screening are consistent with these electronic considerations and support the choice of 55 nm Au NPs for the mechanistic investigation pursued in this study.

## 4 Conclusions

In summary, a systematic and comprehensive experimental investigation of sputtered TiO_2_ ETLs incorporating Au NPs was conducted to elucidate the interplay between structure, interface, and charge dynamics in planar PSCs. The optimized configuration, consisting of a 35 nm double compact TiO_2_ layer with 55 nm Au NPs at 0.15 wt %, led to a 14.2% PCE, outperforming the reference device by 1.3% absolute. The embedded NPs were shown to be fully encapsulated, preserving TiO_2_ crystallinity and electronic structure. Sputtering technique enables precise thickness control and conformal encapsulation of NPs, ensuring reproducibility and scalability in a highly controlled TiO_2_ platform. At low NP concentrations, impedance spectroscopy, intensity-dependent *J*–*V*, and TRPL consistently revealed improved interface passivation and enhanced charge selectivity, attributed to an electron-sink behavior unique to this architecture. This study provides the first systematic demonstration that embedded Au NPs can induce a field-effect modulation of the TiO_2_/perovskite interface through capacitive charge accumulation. At higher NP densities, overlapping depletion regions increased transport resistance, reducing the fill factor and overall efficiency, highlighting the importance of controlled NP concentration for optimal device operation. The electron-sink behavior also mitigated UV-induced photocatalytic degradation of the TiO_2_/perovskite interface, contributing to improved stability during the 25 min illumination period examined in this study. These findings establish sputtered AuNPs@TiO_2_ as a physically robust and electronically tunable ETL platform, offering both practical and conceptual advances toward the design of selective, defect-tolerant oxide interfaces in high-performance perovskite photovoltaics. While sputtered TiO_2_ is employed here as a model ETL, the electron-sink-induced field-effect modulation identified in this work represents a general interfacial concept. Its manifestation in other oxide ETLs, such as SnO_2_, is expected to depend sensitively on band alignment, defect density, and charge-storage capability at the metal NP/oxide interface. Nevertheless, the conceptual framework established here provides a basis for exploring similar electron-sink-driven interfacial modulation strategies in other ETLs.

## Supplementary Material



## Notes

Figure S1: Statistical distribution of the PCE for thicknesses between 10 and 40 nm of the TiO_2_; Figure S2: (α*h*ν)^1/2^ as a function of *h*ν for pristine TiO_2_ and AuNPs@TiO_2_; Figure S3: Spectral dependence of the ellipsometric angles Psi and Delta, for pristine TiO_2_ and AuNPs@TiO_2_ films; Figure S4: Ti 2p and O 1s XPS spectra, for pristine TiO_2_ and AuNPs@TiO_2_ films; Figure S5: XRD diffractograms of pristine TiO_2_ and AuNPs@TiO_2_ films; Figure S6: Statistical distribution of *J*_sc_, *V*_oc,_ and FF for devices with TiO_2_ and AuNPs@TiO_2_; Figure S7: Absorbance spectra of the glass/ITO/TiO_2_/perovskite with pristine TiO_2_ and AuNPs@TiO_2_ films; Figure S8: Schematic of the PSC stack used in FDTD simulations, and calculated *J*_sc_ as a function of NP diameter and interspacing; Figure S9: XRD diffractograms of perovskite on pristine TiO_2_ and AuNPs@TiO_2_; Figure S10: Top-view SEM images of the surface of the perovskite on pristine TiO_2_ and AuNPs@TiO_2_ layers; Figure S11: ln­(*J*_sc_) as a function of ln­(*I*) (PDF)
