# Supplementary material for: Beyond Plasmonics: Au Nanoparticles as Electron Sinks in TiO2 for Interface Passivation Enhancement in Planar Perovskite Solar Cells
Source: ACS Appl Mater Interfaces. 2026 Jan 28;18(5):8321–36. doi: 10.1021/acsami.5c24570 (PMC12903101; doi:10.1021/acsami.5c24570)
Supplement: Supplementary file 1 [file am5c24570_si_001.pdf]

Supporting Information

Beyond Plasmonics: Au Nanoparticles as Electron Sinks in TiO_2 for Interface Passivation Enhancement in Planar Perovskite Solar Cells

Diogo F. Carvalho,*[a,b,c] Pedro Conceição,[a,d] Andrés D. Pardo Perdomo,[a,e] Ricardo Silva,[a] Manuel Martins,[f] Jennifer P. Teixeira,[a] Pedro M. P. Salomé,[a,c] Paulo Fernandes,[a,b,g] Maria Rosário Correia**[b,c]

[a] *INL – International Iberian Nanotechnology Laboratory, 4715-330 Braga, Portugal*

[b] *i3N – Institute for Nanostructures, Nanomodelling and Nanofabrication, University of Aveiro, 3810-193 Aveiro, Portugal*

[c] *Department of Physics, University of Aveiro, 3810-193 Aveiro, Portugal*

[d] *CICECO, University of Aveiro, 3810-193 Aveiro, Portugal*

[e] *Instituto de Energía Solar, ETSI Telecomunicación, Universidad Politécnica de Madrid, 28040 Madrid, Spain*

[f] *PCI - Creative Science Park, 3830-352 Ílhavo, Portugal*

[g] *CIETI, Department of Physics, ISEP – Porto School of Engineering, 4249-015 Porto, Portugal*

*Corresponding author. *E-mail address:* diogocarvalho@ua.pt

**Corresponding author. *E-mail address:* mrcorreia@ua.pt

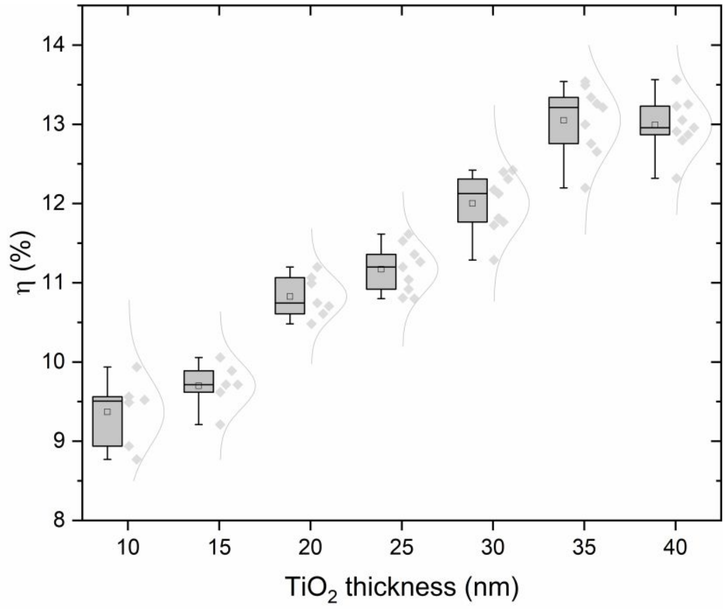

Fig. S1. Statistical distribution of the PCE for thicknesses between 10 and 40 nm of the TiO_2 (measured under reverse-bias scanning). Between 6 and 9 samples were measured for each condition.

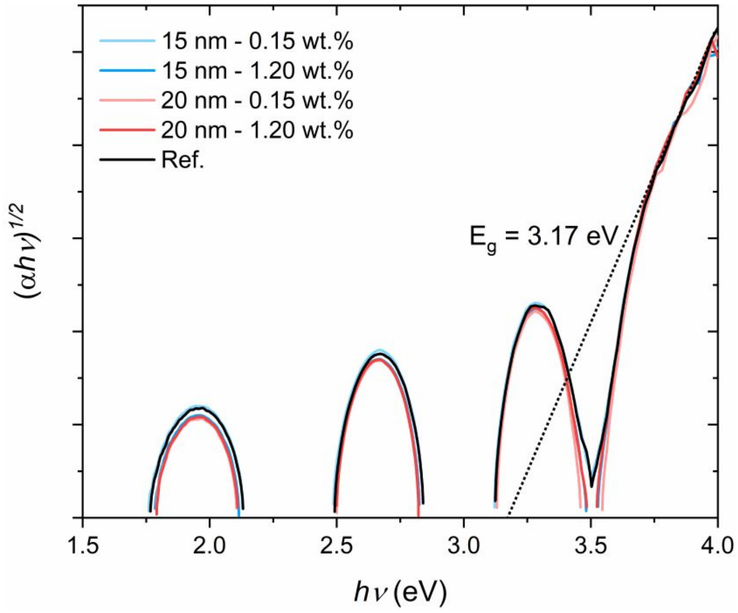

Fig. S2. $(\alpha h\nu)^{1/2}$ (where α is the absorption coefficient and $h\nu$ the photon energy) as a function of $h\nu$ for pristine TiO_2 and AuNPs@TiO_2.

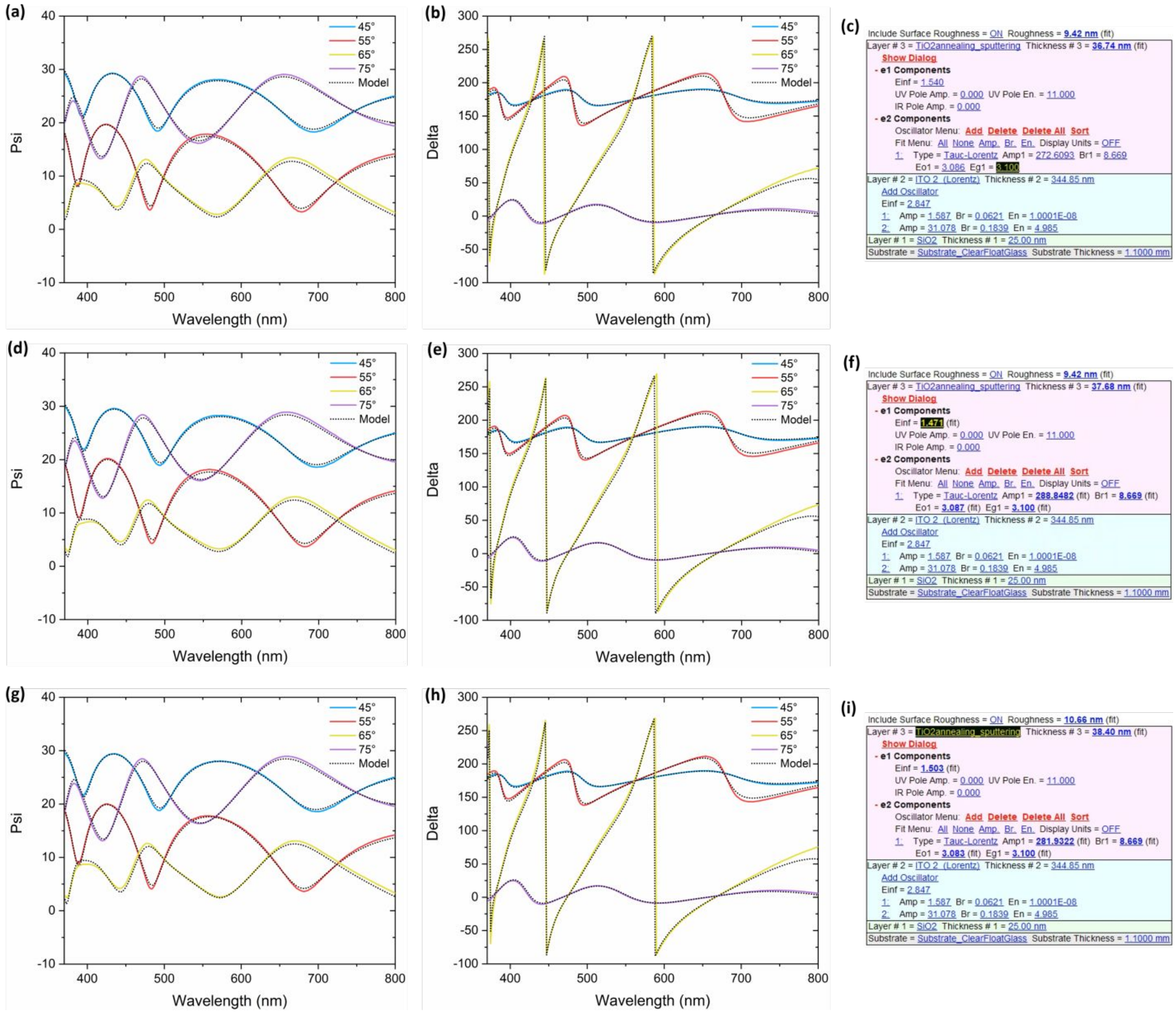

Fig. S3. Spectral dependence of the ellipsometric angles (a), (d), (g) Psi and (b), (e), (h) Delta, for (a)-(c) pristine TiO_2 and TiO_2 films containing NP loadings of (d)-(f) 0.15 wt.% and (g)-(i) 1.20 wt.%, for a 15 nm thickness of the top TiO_2 sublayer. Panels (c), (f), (i) show the model and fitting parameters used in the ellipsometric analysis.

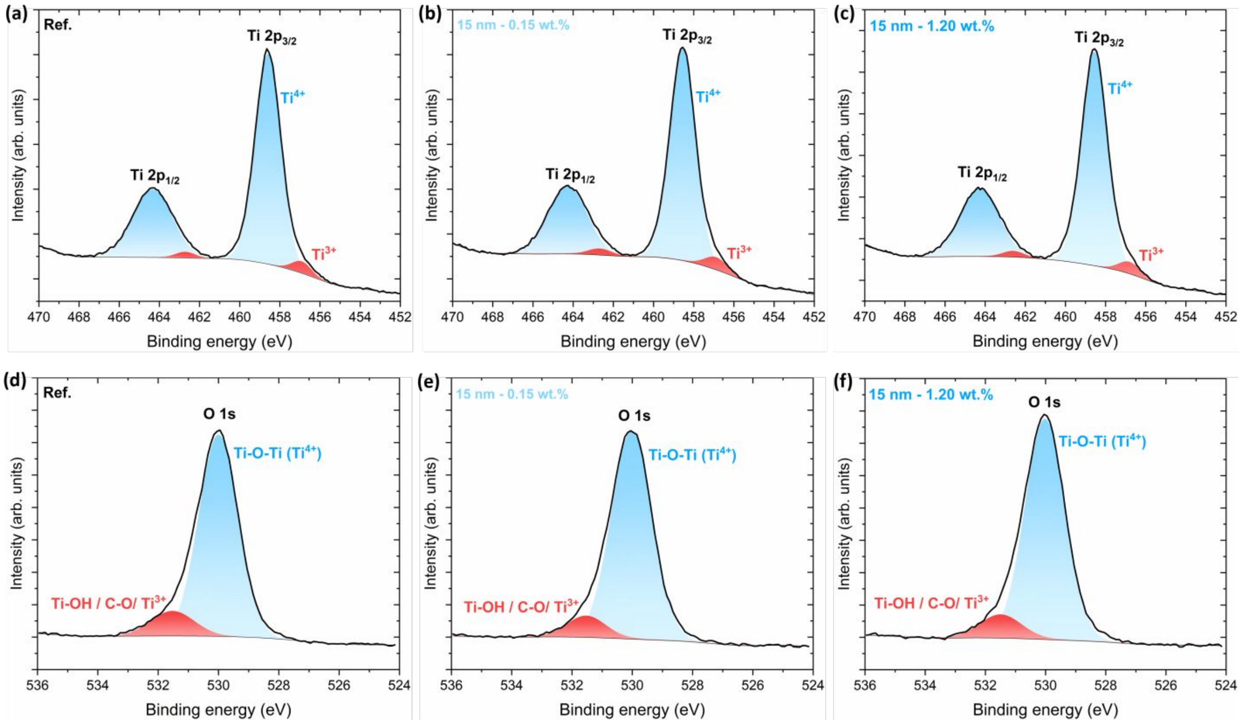

Fig. S4. (a)-(c) Ti 2p and (d)-(f) O 1s XPS spectrum, for (a),(d) pristine TiO_2 and TiO_2 films containing NP loadings of (b)-(e) 0.15 wt.% and (c)-(f) 1.20 wt.%, for a 15 nm thickness of the top TiO_2 sublayer.

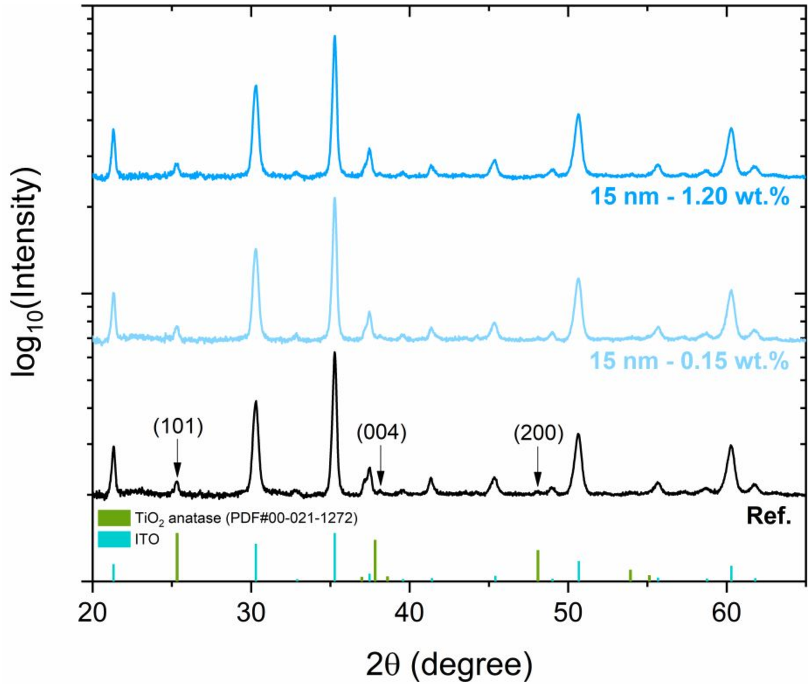

Fig. S5. XRD diffractograms of pristine TiO_2 and TiO_2 films containing NP loadings of 0.15 wt.% and 1.20 wt.%, for a 15 nm thickness of the top TiO_2 sublayer.

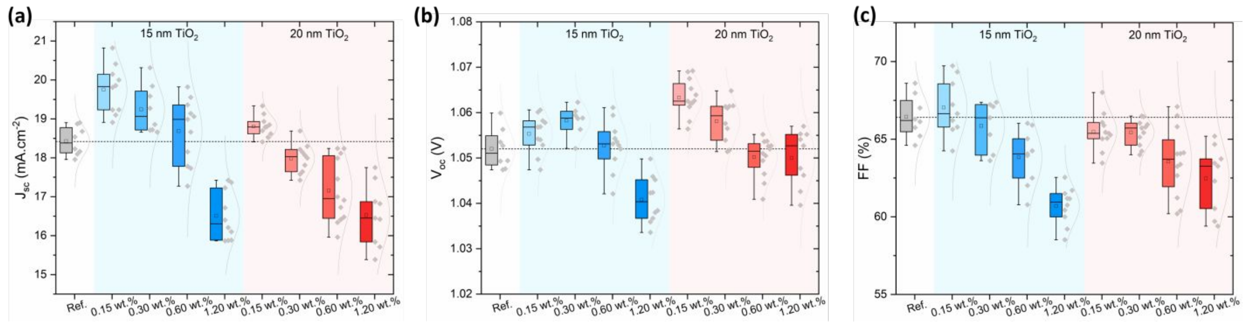

Fig. S6. Statistical distribution of (a) J_{sc}, (b) $V_{oc,}$ and (c) FF for different thicknesses (15, 20 nm) of the top TiO_2 sublayer, for devices incorporating 55 nm Au NPs at concentrations of 0.15, 0.30, 0.60, and 1.20 wt.% (measured under reverse-bias scanning). Between 7 and 12 samples were measured for each condition.

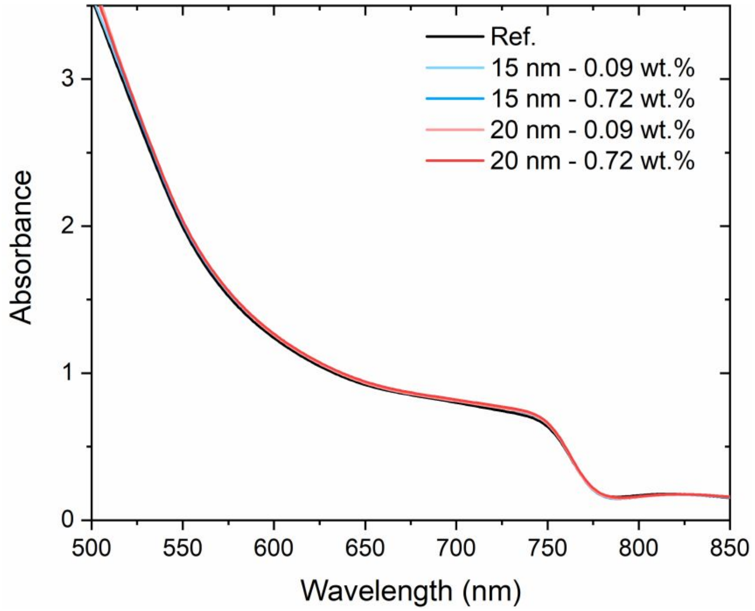

Fig. S7. Absorbance spectra of the glass/ITO/TiO_2/perovskite with pristine TiO_2 and AuNPs@TiO_2 films with top sublayer thicknesses of 15 and 20 nm, and NP concentrations of 0.15 wt.% and 1.20 wt.%.

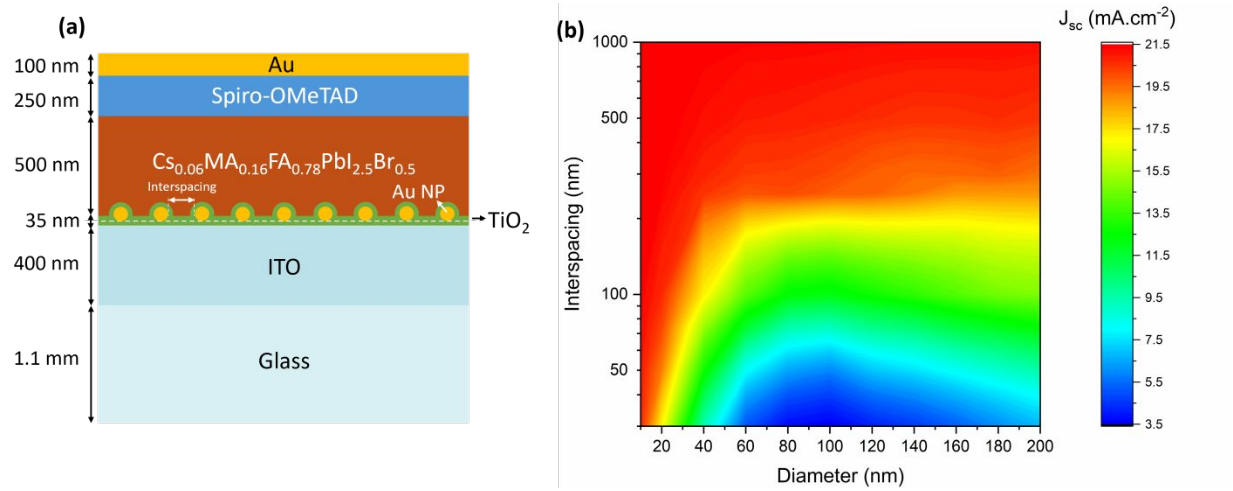

Fig. S8. (a) Schematic of the simulated PSC stack used in FDTD Ansys Lumerical (2021 R2.3), with a 15 nm top TiO_2 sublayer and embedded Au NPs. (b) Calculated J_{sc} as a function of NP diameter (10 – 200 nm) and inter-spacing (30 – 1000 nm; square lattice, surface-to-surface pitch). Refractive indices of glass, ITO, and TiO_2 were estimated by ellipsometry; values for the triple-cation perovskite, Spiro-OMeTAD, and Au were taken from refs. [1–3]. The perovskite volume was kept constant: for each NP configuration, the perovskite thickness was increased by an amount corresponding to the NP volume embedded in the stack. The calculated J_{sc} for the NP-free reference cell was 21.47 mA.cm^{-2}.

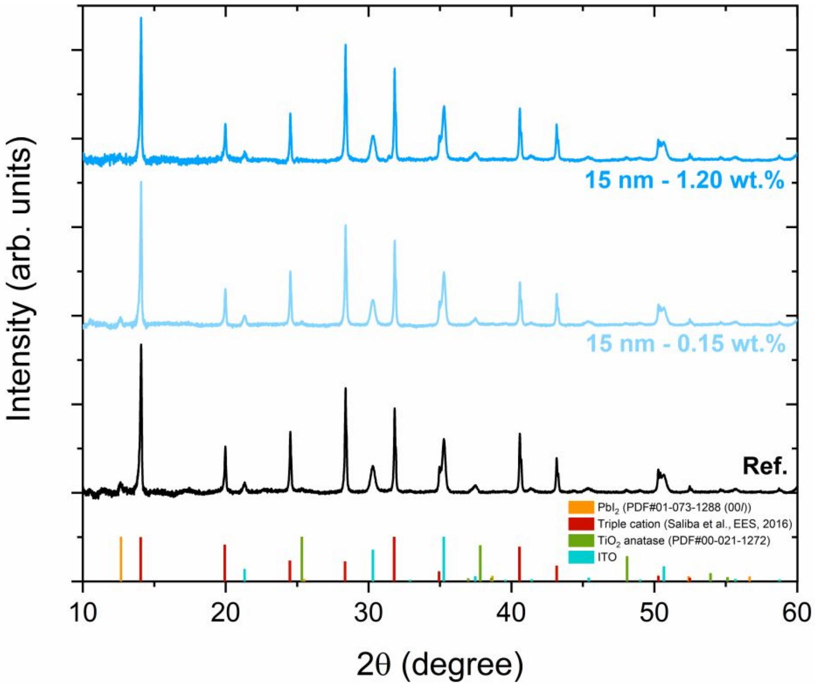

Fig. S9. XRD diffractograms of perovskite on pristine TiO_2 and AuNPs@TiO_2 containing NP loadings of 0.15 wt.% and 1.20 wt.%, for a 15 nm thickness of the top TiO_2 sublayer.

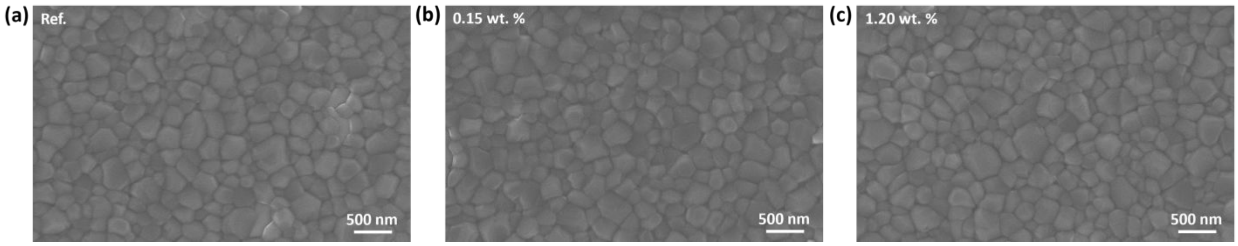

Fig. S10. Top-view SEM images of the surface of the perovskite on (a) pristine TiO_2 and AuNPs@TiO_2 layers containing NP loadings of (b) 0.15 wt.% and (c) 1.20 wt.%, for 15 nm top TiO_2.

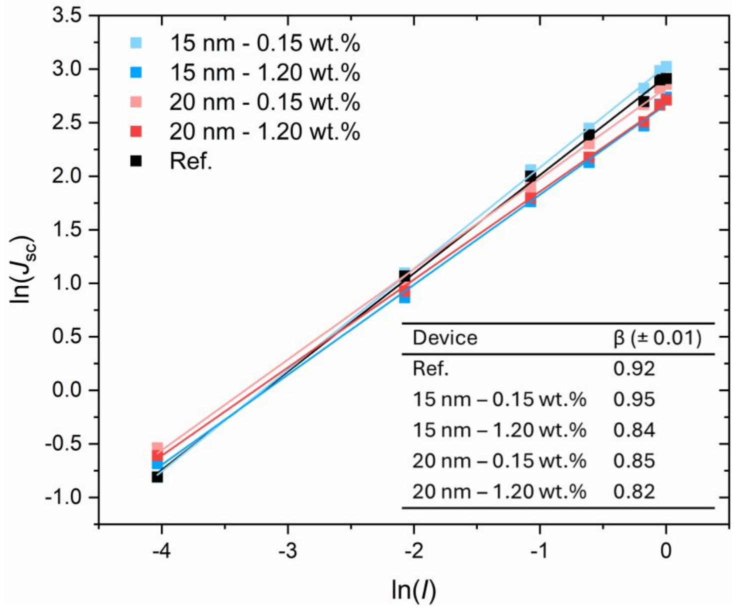

Fig. S11. ln(J_{sc}) (natural logarithm of J_{sc} in mA.cm^{-2}) as a function of ln(I) (natural logarithm of the light intensity in suns), for devices with pristine TiO_2 and AuNPs@TiO_2 with top sublayer thicknesses of 15 and 20 nm, and NP concentrations of 0.15 wt.% and 1.20 wt.%.

References

[1] Y. Feng, Y. Zhang, C. Duan, M. Zhao, J. Dai, Optical properties of CsFAMA-based perovskite film and its application in the inverted solar cells with poly(methyl methacrylate) passivation layer, Opt Mater Express 12 (2022) 3262. https://doi.org/10.1364/OME.463437.

[2] J.M. Ball, S.D. Stranks, M.T. Hörantner, S. Hüttner, W. Zhang, E.J.W. Crossland, I. Ramirez, M. Riede, M.B. Johnston, R.H. Friend, H.J. Snaith, Optical properties and limiting photocurrent of thin-film perovskite solar cells, Energy Environ Sci 8 (2015) 602–609. https://doi.org/10.1039/c4ee03224a.

[3] P. Johnson, R. Christy, Optical Constants of the Noble Metals, Phys Rev B 6 (1972) 4370–4379. https://doi.org/10.1103/PhysRevB.6.4370.
